# Cardiovascular imaging: what have we learned from animal models?

**DOI:** 10.3389/fphar.2015.00227

**Published:** 2015-10-21

**Authors:** Arnoldo Santos, Leticia Fernández-Friera, María Villalba, Beatriz López-Melgar, Samuel España, Jesús Mateo, Ruben A. Mota, Jesús Jiménez-Borreguero, Jesús Ruiz-Cabello

**Affiliations:** ^1^Centro Nacional de Investigaciones Cardiovasculares Carlos IIIMadrid, Spain; ^2^CIBER de Enfermedades Respiratorias (CIBERES)Madrid, Spain; ^3^Madrid-MIT M+Visión ConsortiumMadrid, Spain; ^4^Department of Anesthesia, Massachusetts General Hospital, Harvard Medical SchoolBoston, MA, USA; ^5^Hospital Universitario HM MonteprincipeMadrid, Spain; ^6^Charles RiverBarcelona, Spain; ^7^Cardiac Imaging Department, Hospital de La PrincesaMadrid, Spain; ^8^Universidad Complutense de MadridMadrid, Spain

**Keywords:** animal models, biomedical imaging, heart failure, myocardial infarction, pulmonary hypertension, atherosclerosis

## Abstract

Cardiovascular imaging has become an indispensable tool for patient diagnosis and follow up. Probably the wide clinical applications of imaging are due to the possibility of a detailed and high quality description and quantification of cardiovascular system structure and function. Also phenomena that involve complex physiological mechanisms and biochemical pathways, such as inflammation and ischemia, can be visualized in a non-destructive way. The widespread use and evolution of imaging would not have been possible without animal studies. Animal models have allowed for instance, (i) the technical development of different imaging tools, (ii) to test hypothesis generated from human studies and finally, (iii) to evaluate the translational relevance assessment of *in vitro* and *ex-vivo* results. In this review, we will critically describe the contribution of animal models to the use of biomedical imaging in cardiovascular medicine. We will discuss the characteristics of the most frequent models used in/for imaging studies. We will cover the major findings of animal studies focused in the cardiovascular use of the repeatedly used imaging techniques in clinical practice and experimental studies. We will also describe the physiological findings and/or learning processes for imaging applications coming from models of the most common cardiovascular diseases. In these diseases, imaging research using animals has allowed the study of aspects such as: ventricular size, shape, global function, and wall thickening, local myocardial function, myocardial perfusion, metabolism and energetic assessment, infarct quantification, vascular lesion characterization, myocardial fiber structure, and myocardial calcium uptake. Finally we will discuss the limitations and future of imaging research with animal models.

## Introduction

Cardiovascular disease is the most important cause of mortality in the Western world. It is also responsible for a huge lost in years of healthy life and one of the principal reasons for hospitalizations and emergency room visits. Its epidemiological importance justifies the huge amount of both clinical and experimental research existing in this area. Such research has allowed outstanding therapeutic changes, with impact on patient outcomes. However, not only therapy has improved the outcomes of patients; diagnosis and monitoring tools have improved a lot in recent years. Between these diagnostic tools a special place is reserved for biomedical imaging. Diagnosis, monitoring, follow-up, and research in cardiovascular patients are possible using different imaging techniques.

Using imaging, anatomical, molecular, and functional evaluation is possible in a complete non-invasive way. The progress in cardiovascular patient care has benefited from a rapidly evolving imaging acquisition technique. In the same way imaging would not have developed without the use of animal models. Animal, *in vitro* and *ex-vivo* models are useful for testing hypotheses derived from the clinical setting. They also provide us a scenario in which to evaluate a new imaging tool or tracer. In this review we will discuss the principal animal models used in imaging studies of major cardiovascular diseases.

## Characteristics of animal models for *in vivo* cardiovascular imaging studies

Cardiovascular physiology and diseases are based on the interaction of multiple genes, metabolic processes and the environment, increasing significantly their complexity. These circumstances make highly complicated the full replacement of *in vivo* models by simulated *in vitro* or *in silico* ones. Therefore, animal models for cardiovascular research are pivotal for testing mechanistic hypothesis and for translational research, including the assessment of pharmacological interventions and the development of imaging technologies and surgical devices. In specific fields as drug development imaging techniques application allows to evaluate aspects as target validation, biodistribution, target interaction, pharmacodynamics, and toxicology. Non-invasive, imaging techniques allow multiple measurements to be obtained from a single animal in longitudinal studies. In this way imaging techniques generate significant outcomes using smaller and more efficient experimental designs. This can help to accomplish with the Reduce, Refine and Replace principles in preclinical development processes including experiments with animals. However, the animal model itself is important also in this regard. The selection of the adequate species and covering important aspects as animal manipulation are critical for preclinical drug development success.

### Selection of the adequate animal model

The correct selection of the animal model of cardiovascular research is a great challenge. Bibliography describes plenty of models that mimic the most frequent cardiovascular illnesses. However, in many cases, the authors do not perform a correct comparative anatomy study and the findings do not correlate in the same way that in humans. This issue is especially important for biomedical imaging in which the goal is to identify the aspects that directly interacts or modifies specific anatomical structures. The relative geometry of the heart of each species, the characteristic features of vasculature, muscle mass and conduction system are the main anatomical differences with humans (Hasenfuss, 1998; Hill and Iaizzo, 2009). The choice of the desired model should be made on grounds of etiological induction, animal availability, technological disposal for the species, housing conditions, costs, biological level of study, quality, and quantity of the data, relevance for the human condition and ethical sensitivity (Power and Tonkin, 1999; Hearse and Sutherland, 2000).

#### Rodents and other small mammals

The laboratory mouse is essential in the study of the cardiovascular system. The short gestation period and the low cost of breeding and housing are the main advantages of this species. The knowledge of its genome, the ability to modify it and the rapid data acquisition of genomic modification make attractive the use of mice for studying diverse mechanisms that are affected during the development of cardiovascular diseases (Doevendans et al., 1995, 1998; Bostick et al., 2011). Advances in laboratory animal technology have allowed the miniaturizing acquisition of murine cardiovascular physiology and diagnostic images that define, in a sequential way, the progress of the cardiac illness. However, the mouse shows some obstacles for extrapolation of any outcome of cardiac disease models. Other than animal size and beat (400–600), mouse heart differs from human by: (1) the direct drainage of persistent left superior cava vein into the right atrium; (2) a single opening of the pulmonary vein in the left atrium (Webb et al., 1996; Hoyt et al., 2006); (3) sinoatrial node localization above the junction of right atrium with the cava vein (Meijler, 1985; Hoyt et al., 2006); (4) helicoidal distribution of myocardial fibers (McLean and Prothero, 1992); (5); a large septal branch from the left coronary artery without a proper circumflex branch (still controversial); and (6) the blood support of internal mammary arteries to supply atria, flowing via cardiomediastinal arteries (Michael et al., 1995; Lutgens et al., 1999). Finally, it is important to consider that murine strains and mutations can alter additionally the structure, anatomy, pathology, and physiology of cells, in an unpredictable way and that may change with time (Chien, 1996; James et al., 1998; Kass et al., 1998).

Rat and mouse models show similar advantages, however rats are the classical choice for studying new drug targets in cardiovascular research. The larger physical dimension in rats allows an easier learning of surgical procedures and invasive hemodynamic assessments. The cardiac blood supply originates from both the coronary and extracoronary arteries (internal mammary and subclavian arteries), but the principal limitations are focused in myocardial function: a short action potential which normally lacks a plateau phase, and α-myosin heavy-chain isoform predominates with β-myosin isoform shift under hemodynamic load or hormonal condition (Swynghedauw, 1986; Hasenfuss, 1998; Bers, 2001).

Larger species as rabbits and dogs show a higher similarity with human heart and allow the study of the left ventricular function in models of heart failure. Like humans, in these two species, β-myosin heavy-chain predominates and excitation–contraction coupling processes seem to be analogous to those in the human myocardium (Lompre et al., 1981; Hasenfuss, 1998). However, canine heart presents a dense collateral coronary branching with a higher proportional relation of its size with respect of thoracic cavity. Besides, the proportion of heart to bodyweight is near the double of the human (Verdouw et al., 1998).

#### Large animal models

Direct translation from rodents to humans has to be taken with caution because of the species-related differences, such as contractility, architecture, heart rates (HRs), oxygen consumption, protein expression, etc… (Zaragoza et al., 2011). Instead, large animal models have a better translational bridge between preclinical and clinical studies because of their anatomical and physiological similarities (Fernández-Jiménez et al., 2015a). Predominately, swine species are the election in preclinical cardiovascular research. Their anatomical heart features resemble those described in humans: coronary arteries support a blood flow with a right-side dominant circulation to the conduction system from the posterior septal artery, and less subepicardical anastomosis than in other species such as the dog; the electrophysiological system is more neurogenic than myogenic with prominent Purkinje fibers; the aorta has a true vasa vasorum network like that of humans; and hemodynamic values that allow the extrapolation and translation of reliable experimental data (Verdouw et al., 1998; Unger, 2001; Laber et al., 2002; Swindle, 2007; Lelovas et al., 2014). On the contrary, pigs show a left azygous (hemiazygous) vein which drains the intercostal vessels into the coronary sinus instead of precava, and the endocardium and epicardium are activated simultaneously because of differences in distribution of the specialized conduction system in the ventricles (Swindle, 2007; Lelovas et al., 2014). The principal drawbacks of experimentation with pigs are the high cost of housing and care, especially in heart failure models. Also changes that occur during animal growth are particularly important for translational imaging research, specially the change in the proportional heart weight to bodyweight ratio. That ratio for a 25 Kg-farm pig is 5 gr/Kg (as human) and this proportion is kept in juvenile animals, and decreases significantly when the pig reaches the sexual maturity (Verdouw et al., 1998; Lelovas et al., 2014). Nowadays, the advent of miniature species like Yucatan, Hanford, Sinclair, and Göttingen minipigs has significantly solved both limitations (Bode et al., 2010).

Other species, like the sheep show morphologic similarities with humans in regard to adult heart size, venous drainage and physiological responses during the induction of cardiovascular diseases; thus, sheep allows an experimental scenario highly used and reliable in biomedical imaging studies. Among the differences with human anatomy, a left-dominance coronary artery support (but with a lack of preformed collateral circulation), the absence of intervalvar septum, the valvular “os cordis,” aortic valves fragility and the left thoracic drainage of the azygous vein directly to coronary sinus, are the most noticeable. Nevertheless, the main limitations of ovine models are the risk of zoonotic diseases and their condition of ruminant, whose features of the stomach could interfere in some non-invasive image acquisitions (Walmsley, 1978; Dixon and Spinale, 2009; Hill and Iaizzo, 2009).

### Methodological considerations

The ideal scenario for any imaging acquisition conducted in cardiovascular disease animal models is the one performed with awake and cooperative animals. However, this requires acclimatization to restraint to reduce distress as confounding variable. Conversely, each model under normal sleep conditions is preconditioned by the type and doses of anesthesia and, in lesser extent, analgesia used in the experiments. The correct selection of the anesthetic protocol will determine the reliability and interpretation of the measured data. All the anesthetics induce a direct or indirect depression of hemodynamic values and cardiac functionality. Injectable drugs can create an adequate level of unconsciousness to perform the injury required in the model and permit a complete imaging study. But, the hemodynamic response to the same drugs is different between species which depends on their metabolic features. This can hinder an stable imaging acquisition. For example, rodents generally require 3–5 times the doses used for large animals. This is critical in those surgical models that imply an open-chest approach, which means a great impact in the animal thermoregulation and the size of the infarct area, anesthesia timing, cardiovascular depression, etc… Due to their minimal systemic metabolism and a short recovery phase, inhaled anesthetics offer more security for the development of the procedures; nevertheless, inhalation agents like sevofluorane or isofluorane protect the myocardium against the insult of the hypoxic states and diminish significantly the immune cellular transmigration on inflammatory injuries (Rao et al., 2008; Ge et al., 2010; Chappell et al., 2011).

During the imaging studies, anesthesia is crucial to maintain the animal within stable, well-defined physiologic parameters which is indispensable for detecting critical pathophysiologic responses associated to the cardiovascular disease model. Not only the primary affection that is provoked in the animal model but also variables related with the animal homeostasis in response to such affection should be taken into account. Also other indirect factors as risk of hypothermia (and the autonomic response to it) during surgical procedures can affect the results of cardiovascular imaging studies. Anesthesia affects the blood flow, blood oxygenation levels, and cardiac and respiratory functions, which should be correctly monitored, especially in those modalities involving long acquisition times. Inhaled agents are eliminated quicker via the lungs, whereas injectable agents need to be metabolized by the liver and excreted by the kidneys. Both sevofluorane and isoflurane are minimally metabolized by the liver and increase the efficiency of organ perfusion. This means a less toxic effect to the animal metabolism, rapid induction, minor impact on cardiovascular function and quick recovery, which make them in many cases the choice for imaging studies. Indeed, isofluorane has been described as the election for PET studies in experimental cardiology just for the improvement of cardiac radiotracer uptake vs. injectable anesthetics (Gargiulo et al., 2012a; Lee et al., 2012).

Another key point is the selection of a determined animal gender. It is has been described that males tend to develop an eccentric hypertrophy and left ventricular dilatation in certain cardiovascular disease, whilst females show a more concentric hypertrophy with a better preserved left ventricular function (Mahmoodzadeh et al., 2012). Several studies with rodents define a protective role of the female hormone 17-β-estradiol and its respective estrogen receptors mediating the cardiovascular responses to different pathophysiological situation. This steroid regulates the expression of a variety of dependent genes related to myocyte cytoskeletal proteins, cell-to-cell interaction, Ca^2+^ channels and apoptosis inhibition (Patten et al., 2004; Groten et al., 2005; Mahmoodzadeh et al., 2010, 2012). For that reason, females tended more easily to develop a ventricular hypertrophic response against extreme effort stimuli to preserve the heart outflow, and showed significantly smaller infarct area after ischemic myocardial conditions or even maintained the heart morphology and functionality under pressure overload stimuli (Wang et al., 2005; Johnson et al., 2006; Babiker et al., 2007; Patten et al., 2008; Foryst-Ludwig et al., 2011; Mahmoodzadeh et al., 2012).

## Imaging in myocardial infarction and coronary artery disease

Myocardial infarction (MI) may be the first manifestation of coronary artery disease (CAD) that is the number one cause of death among adults (Lloyd-Jones et al., 2010; Nichols et al., 2014). Myocardial tissue-specific biomarkers and high sensitive imaging techniques allow MI definition as any amount of myocardial injury or necrosis in the setting of myocardial ischemia (Thygesen et al., 2012). Animal models on MI are essential for the better understanding of CAD, for discovering risk biomarkers of MI, for studying early diagnostic test, and also for establishing beneficial effects of new therapies.

### Small and large animal models for MI assessment: mouse and porcine models

In small animals, including mice and rats, the left coronary artery ligation procedure developed by Pfeffer et al. (1979) is the most common method used to induce acute myocardial damage. The artery might be either permanently or temporary occluded to reproduce human ischemia/reperfusion injury. In respect to large animals, swine is the preferred animal model of heart damage, because of the absence of collateral coronary circulation, similar arterial anatomy compared to humans and the suitability to have clinically relevant imaging techniques to accurately quantify area at risk or infarcted tissue (Crick et al., 1998). One of the most widely used model of MI in pigs is the angioplasty balloon occlusion of the left anterior descending coronary artery (Ibanez et al., 2007). Moreover, the development of gene-engineered animals with the advent of molecular genetic techniques during the last years has allowed an explosion in the number of models resulting in a tremendous progress in the understanding of myocardial diseases.

### Different non-invasive imaging modalities to assess myocardial structure and function, inflammation, and viability in animal models of MI

#### Myocardial structure and function: techniques and current evidence

Two-dimensional echocardiography is a well-established tool that has have been largely used for the assessment of cardiac function and structure using techniques and indices familiar from human echocardiography. It offers a rapid and low cost evaluation of heart anatomy, function and biomechanics (Richardson et al., 2013) and response to treatment (Bao et al., 2013; Matthews et al., 2013). But, its results depend on the body complexion, echographic window and the HR of the scanned animal. As a result, ultrasound based methods have been mostly limited to small-animal models these days. The advent of higher frame rates and smaller probes operating at higher frequencies equipment's have facilitated imaging of mice, a setting where CMR is still challenging. Basic measurements of LV systolic function, LV mass, and LV chamber dimensions are easy to achieve from a parasternal long and short-axis view of the heart. In the absence of wall motion abnormalities, M-mode is an accurate method for evaluating LV structure and function using the Teichholz method for fractional shortening (FS%) and ejection fraction (EF%) estimation (Tanaka et al., 1996). However, in mice models of MI, wall motion abnormalities and systolic function should be determined in 2D mode echocardiography with consecutive parasternal short-axis planes using the Simpson's rule (Gao et al., 2000). It is important to note that marked changes in echographic measurements occur when mice are anesthetized (Rottman et al., 2003). Anesthesia depresses HR, and the FS% is directly affected by cardiac frequency. Thus, it is important when protocols required consecutive measurements to perform all echoes under the same conditions. Regarding diastolic dysfunction, it is a critical condition where blood filling of the LV is impaired. It accompanies, and sometimes precedes many disease conditions like ischemic heart disease, but it is more difficult to define and to measure than systolic dysfunction. Moreover, its echographic measurement are highly affected by loading conditions, age and HR. Transmitral filling, alterations in the A-wave or the E/A ratio haven been used to define diastolic abnormalities. However, E and A waves are fused due to rapid HR in mice, so as, other indices like color M-mode flow propagation velocity (Schmidt et al., 2002), isovolumentric relaxation time, the A'-wave or the E'/A' ratio using tissular Doppler imaging (Schaefer et al., 2003, 2005), also the Tei index (Tei et al., 1995) that characterize global, systolic and diastolic left ventricular function after MI, have been used in mice. However, the accuracy with which these measurements quantify diastolic dysfunction is still open to discussion (Scherrer-Crosbie and Thibault, 2008).

Cardiac Magnetic Resonance (CMR) is up to date the preferred technique for the assessment of cardiac morphology and function in animal models (Stuckey et al., 2008; Makowski et al., 2010). CMR provides non-invasive high image quality tomographic views of the heart with sub-millimeter anatomical detail, high tissue contrast and excellent reproducibility, which can be used for accurate functional and structural assessment in coronary heart disease (Sinitsyn, 2001). They have been used to serially evaluate left and right ventricular dysfunction.

Medium-large animal models can be studied with conventional procedures established in the clinic for patients, but imaging small animal models with CMR is challenging due to their faster HR and smaller dimensions of the heart, and requires the use of high-field (>4.7T) scanners or substantial modifications of conventional protocols used in clinical 1.5 or 3T platforms (Gilson and Kraitchman, 2007; Bunck et al., 2009). Cine sequences in Magnetic Resonance Imaging (MRI) are used to study all relevant functional parameters of the left ventricle (LV) such as EF, ventricular volume, cardiac mass, and cardiac output with high accuracy (Franco et al., 1999). Several ECG-gated spin-echo and multiphase gradient-echo (cine MRI) have been developed for quantifying LV parameters in mice with similar reliability (Ruff et al., 1998; Slawson et al., 1998) becoming the gold standard for LV assessment in rodents (Slawson et al., 1998; Franco et al., 1999). Also, high in-plane resolution (0.1 × 0.1 mm) cine MRI has been developed to quantify right ventricular function in murine models (Wiesmann et al., 2002). Other techniques for quantitative wall-motion imaging like myocardial tagging (Epstein et al., 2002; Zhou et al., 2003) or velocity-encode phase-contrast imaging (Espe et al., 2015) have been optimized for animal studies. These approaches permit the tracking of regional myocardium and enable the quantification of principal strains and directions (radial, circumferential, and longitudinal) to depict the extent of the changes in contractility after MI (Young et al., 2006).

**Figure 1:**
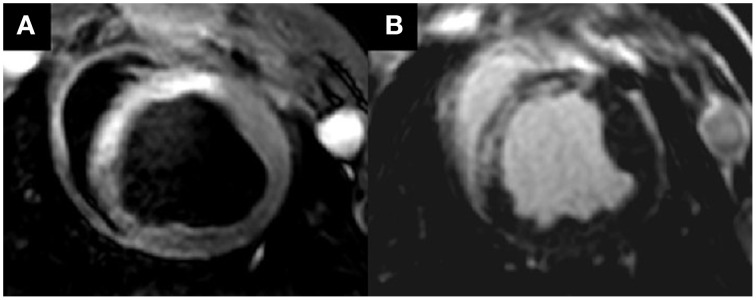
**Cardiac magnetic resonance images of an anterior acute myocardial infarction in a pig model of ischemia/reperfusion injury**. **(A)** Area at risk in T2-STIR sequence and hyperintense zone in anterior septum. **(B)** Necrotic zone in late enhancement sequence at the same zone. Published with publisher's permission. Original source: Fernández-Friera et al. (2013). Copyright © 2012 Sociedad Española de Cardiología. Publicado por Elsevier España, S.L. All rights reserved.

Myocardial ischemia also leads to a variety of changes in tissue structure. Myocardial fibrosis is the main structural damage after ischemia/reperfusion injury. Scar tissue can be evaluated with inversion recovery echo-pulse sequences for late gadolinium enhancement to differentiate between reversible damage and infarcted myocardium after MI (Figure 1; Kim et al., 1999). Also, diffuse microfibrosis can be detected in the myocardium using recent T1-mapping sequences in animals (Stuckey et al., 2014; García-Álvarez et al., 2015). Nevertheless, efforts are still required to further improve and standardize protocols and to generate reference values for each animal model on cardiovascular disease.

#### Myocardial inflammation: area at risk: techniques and current evidence

**Figure 2:**
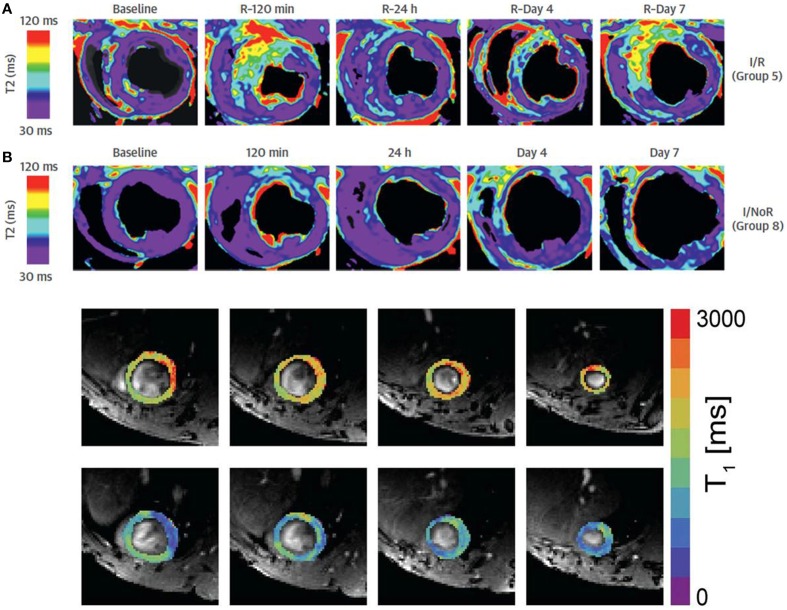
**Examples of T2 (A) and T1 parametric maps in animal models of myocardial infarction**. **(A)** Shows the dynamic changes in T2 relaxation times in the ischemic region after permanent coronary occlusion and reperfusion in a pig. **(B)** Shows pre contrast (upper row) and post-gadolinium contrast on a mouse model. Adapted from Figure 5 of Fernández-Jiménez et al. (2015a) and from Figure 2 of Coolen et al. (2011) with original publisher's permission (Bio Med Central).

The area at risk (AAR), defined as the hypoperfused myocardium during an acute coronary occlusion, is a major determinant of infarct size and clinical outcomes in MI. Additionally, accurate AAR quantification is important because it has been used as an end-point in clinical trials testing the efficacy of cardioprotective interventions (Feiring et al., 1987). Single-photon emission computed tomography (SPECT) is the traditional reference method for determining AAR by injection of technetium-based tracer before opening of the occluded vessel. However, CMR has been proposed as an alternative approach over the last years because of higher spatial resolution and the absence of tracer administration need or radiation exposure (Carlsson et al., 2009). In particular, T2-weighted (T2W) edema-sensitive sequences have enabled the identification of acutely ischemic myocardium by detecting increased signal intensity that reflects myocardial water content (Aletras et al., 2006; Friedrich et al., 2008). T2W imaging, however, is often limited by motion artifacts, incomplete blood suppression, low signal-to-noise ratio and coil sensitivity related-issues of surface coil. Newer quantitative CMR methods include: (1) T1 and T2 mapping imaging (Figure 2) that allow direct measurement of intrinsic tissue properties. These sequences are less dependent on confounders affecting signal intensity (Ugander et al., 2012) and their accuracy for AAR quantification is high compared to microsphere blood flow analysis in a dog model of ischemia/reperfusion injury (Fernandez-Jimenez et al., 2015b); (2) BOLD or modified blood oxygen level-dependent sequences which have been recently proposed to detect ischemic myocardium in a dog model of severe coronary stenosis (Tsaftaris et al., 2013); (3) Targeted microparticles of iron oxide, which shorten T2 and T2^*^ relaxation times. By tracking up-regulated vascular cell and intercellular adhesion molecules, such as VCAM and ICAM, it is possible to detect and localize myocardial ischemia (Grieve et al., 2013); (4) Balanced steady-state free precession sequences with T2 preparation have also been proposed to detect myocardial edema in a porcine model and in patients with reperfused acute MI (Kellman et al., 2007).

In addition to CMR approaches, pre-reperfusion multidetector Computed Tomography (CT) imaging has also been described as a method to assess AAR size in a porcine acute MI model (Mewton et al., 2011).

### Myocardial perfusion and viability by PET and MRI

Positron Emission Tomography (PET) currently plays an important role in clinical cardiology (Bengel et al., 2009). The basic principle of PET is the coincidence detection of the annihilation photons emitted after the emission of a positron by a beta+ radioisotope. The spatial resolution of PET images is currently in the range of 4–7 mm for clinical scanners and about 1 mm for small animal systems. Higher detection sensitivity allows measuring radiotracer at nano- to pico-molar concentrations. In addition, PET is a truly quantitative imaging tool that measures absolute concentrations of radioactivity in the body and allows for kinetic modeling of physiologic parameters such as absolute myocardial blood flow or glucose use. The data acquisition can be synchronized with an ECG or respiration signal and retrospectively used to obtain gated images. PET systems are nowadays combined with CT systems that offer fused anatomical and functional images. Combined PET and MRI systems have recently appeared as and attractive option but their use is still mostly limited to research studies (Nekolla et al., 2009).

Cardiac imaging in small animals is challenging due to the small ventricle volume and wall thickness and the high HR (Gargiulo et al., 2012b). Dedicated small animal PET systems with high spatial resolution and increased sensitivity have been developed (Levin and Zaidi, 2007). In the other hand, large animal models are typically imaged in clinical systems (Teramoto et al., 2011). In planning longitudinal PET studies with animals, many variables interfering with the accuracy of the experimental results must be taken into account (Adams et al., 2010; stress related to physical restraint, fasting, warming and anesthesia).

#### Myocardial perfusion

Myocardial MRI based techniques are based (in large animal models and in humans) on regional differences in myocardial signal intensity during the first passage of an intravenously administered dual-bolus of gadolinium-based contrast agent, although quantitative approaches systemically underestimate myocardial reserve and require many manipulations and have limited inclusion in the clinical routine. For the successful application of these methods in mice, imaging technology requires the complete acquisition of imaging dataset at every single or every second heartbeat using non-conventional MRI pulse sequences. MRI offers the advantage by comparison with nuclear medicine-based techniques of high resolution and consequently betters assessment of transmural perfusion. MRI perfusion technology has also the advantage that can be integrated in routine CMR protocols of functional assessment and late gadolinium enhancement. Most of the semi-quantitative methods are based on the contrast enhancement ratios or upslope indexes, although these systematically underestimate myocardial perfusion. Finally, recent alternatives for absolute quantification included dual saturation strategies of single bolus acquisition, that will make easier to implement in clinical protocols (Sánchez-González et al., 2015).

Alternatively, other MRI based—Arterial Spin Labeling (ASL) perfusion methods does not use an exogenous contrast agent and has been used for single slices to measure and quantify myocardial perfusion also in small animals, although with long acquisition times (Kober et al., 2005). ASL uses the water of the blood as endogenous tracer and allows *in vivo* quantification of the absolute perfusion and the regional blood volume in the myocardium. These methods showed good agreement with standard *ex vivo* microspheres technique and is sensitive enough to detect and visualize regional alterations of the perfusion after MI (Streif et al., 2005).

Basic experiments based in nuclear medicine have provided complement or additional information to MRI based methods. Myocardial perfusion imaging with PET is a standard tool for detection of CAD, risk stratification of patients, and guidance of therapeutic interventions (Di Carli et al., 2007). Regional blood flow at rest may be normal until the stenosis is higher than 90%. However, autoregulation is incapable of preserving maximum blood flow during exercise or pharmacological stress test leading to reduced myocardial blood flow relative to demand and stress-induced ischemia. Thus, in a patient with coronary artery stenosis, when acute myocardial ischemia occurs, the initial abnormality is an imbalance in blood flow between the hypoperfused and normally perfused areas (Di Carli et al., 2007).

CT coronary angiography is considered the gold standard for evaluating the presence and the severity of coronary stenosis, which provides the anatomical extent of disease. However, perfusion imaging provides hemodynamic significance of epicardial stenosis. PET myocardial perfusion imaging combined with tracer-kinetic modeling can provide absolute quantification of regional myocardial blood flow of the LV. Tracer kinetic modeling requires dynamic imaging beginning briefly before the tracer injection and monitoring of tracer distribution in the myocardium for 2–30 min depending on the tracer and model. Rest and stress scans are typically performed sequentially and stress scans in animals are achieved by pharmacological stress by infusion of adenosine, dipyridamole, or dobutamine.

**Figure 3:**
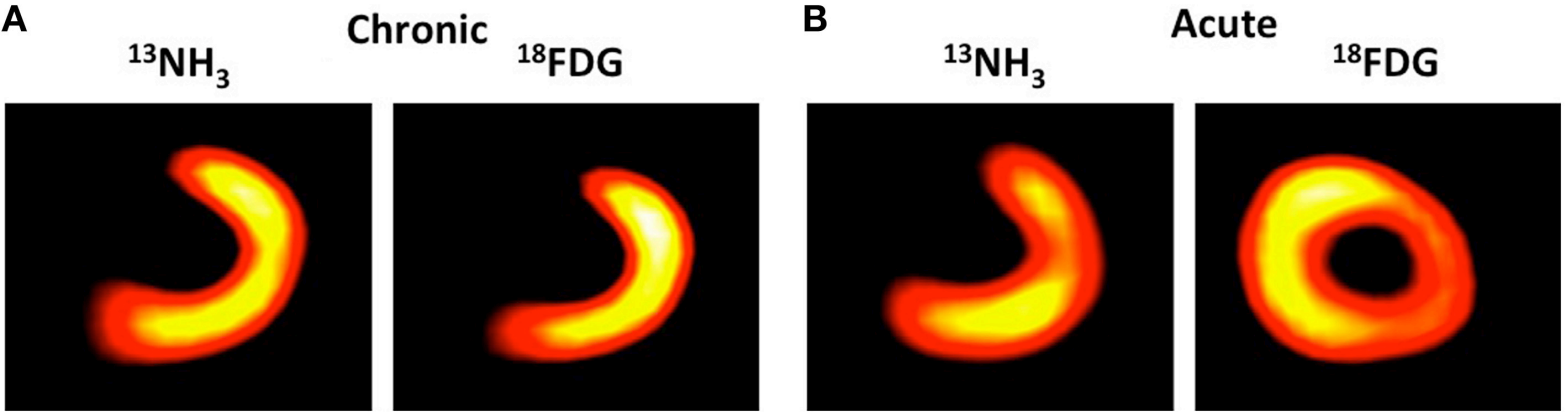
**Representative midventricular short-axis slices PET myocardial perfusion at rest and PET myocardial metabolism using ^13^NH_3_and 18FDG tracers respectively in pigs studied in the chronic (A) and acute (B) phases after myocardial infarction**. From Lautamäki et al. (2009) Figure 1. With permission.

The available flow agents are characterized by a rapid myocardial extraction and by a cardiac uptake proportional to blood flow. PET radiotracers used for evaluation of myocardial blood flow include ^13^NH_3_(see Figure 3), ^82^Rb, and H${}_{2}^{15}$O. However, their short half-life limits their widespread clinical use, because of the need for nearby cyclotron (^13^N and ^15^O) or generator (^82^Rb). Other agents based on ^18^F as Flurpiridaz (Packard et al., 2014) shows potential to spread the use of PET for cardiac perfusion imaging, even with animal models, as it does not depend on onsite cyclotron or generator.

#### Myocardial metabolic and energetic viability

Alterations in myocardial substrate metabolism are critical in the pathogenesis of many cardiovascular diseases. MI is associated with numerous biochemical and functional changes in the necrotic tissue, in the AAR, and in the remote myocardium. ^18^F-fluorodeoxyglucose (FDG) is a glucose analog that is widely available due to its success as a metabolic imaging tracer in clinical oncology. FDG traces myocytic glucose uptake and can be used to quantify regional myocardial glucose metabolism (Dilsizian et al., 2009; see Figure 3). FDG is employed to determine the extent of myocardial viability or potentially reversible contractile dysfunction in response to revascularization as well as the extent of scar tissue or irreversible contractile dysfunction. Increased FDG uptake can be observed in ischemic tissue while significantly reduced or absent uptake indicates scar formation.

The diagnostic quality of the myocardial FDG image depends on the concentration of tracer in both myocardium and blood. Myocardial FDG uptake depends quantitatively on plasma concentrations of glucose and insulin, the rate of glucose utilization and the relationship between FDG and glucose defined by the lumped constant. High plasma concentrations of glucose lower the fractional utilization of FDG and thus degrade the quality of myocardial FDG uptake images. Myocardial glucose uptake also depends on myocardial work, plasma levels of free fatty acids, insulin, catecholamines, and oxygen supply. FDG uptake is heterogeneous in normal myocardium in the fasting state. Therefore, attempts have been made to standardize the metabolic environment for human myocardial FDG imaging (Knuuti et al., 1992), whereas procedures for animal imaging vary widely (Gargiulo et al., 2012b). In humans, patients are studied under fasting conditions following oral glucose loading or during hyper-insulinaemic-euglycaemic clamping to improve image quality and diagnostic accuracy.

Alternatively, Magnetic Resonance spectroscopy (MRS), primarily based in 31P and 1H provides an energetic profile (ATP, PCr, etc…) or lipid content, respectively that in are both connected to the possible change in myocardial substrate utilization from fatty acid toward glucose in the context of myocardial ischemia/reperfusion injury or provides useful insights into the effects of obesity on the heart. These methods have sometimes used to evaluate the improved cardiac energetic and function effect of novel drugs (Bao et al., 2011). 31P MRI is the only method available to provide non-invasive measures of endogenous quantitative concentration of these energetic metabolites and creatine kinase kinetics (Bottomley et al., 2009). Additionally, 13C in natural isotopic abundance is *per se* not very informative. However, possible hyperpolarization with an SNR increase in five orders of magnitude is the technique with promising future for enhanced 13C MRS studies, particularly of the glycolytic metabolism of the heart. There are some reports of 13C metabolic images of lactate, alanine, bicarbonate and pyruvate in a pig heart following coronary occlusion (Bottomley et al., 2009).

## Heart failure

A variety of animal models have been used to mimic the human disease with the highest interest in medical cardiology (Table 1; Verdouw et al., 1998; Dixon and Spinale, 2009; Patten and Hall-Porter, 2009; Abarbanell et al., 2010; Houser et al., 2012). HF is caused by an unable heart to maintain oxygen level that vital organs demand deriving from an impaired blood filling and/or ejection (Houser et al., 2012). According to these two different causes, there are two phenotypes of HF, ones derived from the systolic dysfunction, where the EF decreases below 50%, called HF with reduced EF; and ones derived from a diastolic dysfunction, where the EF remains above 50%, called HF with preserved ejection EF. Valve diseases, hypertension, myocardial ischemia and genetic abnormalities that caused dilated and restrictive cardiomyopathies are the most common mechanisms used for creating experimental HF. Characterization of animal models of HF requires revealing an insufficient cardiac output, on one hand the left-, right-, or biventricular cardiac dysfunction, and o pulmonary findings compatibles with the course of HF (Houser et al., 2012).

**Table 1:** **Schematic comparison of the different animal models used in cardiovascular imaging research**.

**Specie**	**Model**	**Failure etiology**	**Advantages**	**Disadvantages**
**CARDIAC HYPERTROPHY**				
Mouse	Transverse aortic and pulmonary artery constriction	Acute and pressure overload	Easy use of GEM animals. Hypertrophy developed rapidly (2–3 weeks)	Surgical skills. Acute hypertension and expense of equipment for cardiovascular imaging and physiology assessment
Mouse	Isoproterenol infusion	Toxic injury of myocardium	Minimal surgery and good scenario for pharmacological or gene therapy	Hypertrophy is adjusted to dose and mouse strain
Rat	Spontaneous hypertensive rat and Dahl salt-sensitive rat	Chronic pressure overload	The onset of hypertension is gradual, being the heart failure in later stages. Genetic origin of hypertension. No surgery	Long experimental period (6–12 months)
Rat	Ascending aortic and pulmonary artery constriction	Gradual to quick onset pressure overload	Gradual to quick onset hypertension	Less GEM animals and similar cost of equipment for cardiovascular physiology assessment than mouse
Rat	Arteriovenous shunts	Overload of ventricular chambers	Progressive heart hypertrophy, more rapidly in the right ventricle. Well tolerate and it possible to reverse the volume-overload state	Greater surgical skills, with a grade of hypertrophy fistula localization-dependent
Guinea pig	Descending aortic constriction	Pressure overload and hypertension	Human mimicking alteration of sarcolemma calcium handling	Special and expensive requirements for husbandry
Rabbit	Aortic and pulmonary constriction	Gradual onset pressure overload	Imaging technology allows normalizing the grade of constriction. Possibility to reverse the pressure-overload situation	Thoracotomy surgery required
Rabbit	Doxorrubicin	Toxicological aggression	Myocyte function and structure modification	High risk of mortality dose dependent
Dog	Aortovenus shunt	Volume overload	Progressive heart hypertrophy, more rapidly in the right ventricle	Not so well tolerated than rats. Frequent arrhythmias, edema and quick health decrease
Dog	Arrhythmogenic right ventricular cardiomyopathy of Boxer	Desmosomes proteins mutation	Genetic origin which mimic the human disease	Social ethical considerations
Cat	Inherited Hypertrophic Cardiomyopathy of Maine Coon and Persian strains	Sarcomeric protein gene mutations	Genetic origin which mimic the human disease	Social ethical considerations
Pig	Descending aortic constriction	Pressure overload and hypertension	Progressive hypertrophy and animal well adapted (constriction grade progresses with animal growth)	Surgical skills and lateral thoracotomy
Pig	Pulmonary artery hypertension by microembolization	Increased vascular resistance	Progressive hypertrophy of right ventricle and final heart failure by dilated cardiomyopathy. No surgery	Great hypoxic vasoconstriction
Sheep	Ascending aortic constriction	Pressure overload and hypertension	Transition from compensated hypertrophy to left ventricular dysfunction	Zoonotic risk
Sheep	Pulmonary artery hypertension by microembolization	Increased vascular resistance	Progressive hypertrophy of right ventricle and final heart failure by dilated cardiomyopathy. No hypoxic vasoconstriction No surgery	Zoonotic risk
**DILATED CARDIOPATHY**				
Mouse	Genetic Engineering modified animals (GEM)	Dilated cardiomyopathy	Genetic modifications of structural and functionality of cardiomyocytes. No required surgery	Clinical reliability restricted to the molecule of study: e.g., TNF-α overexpression
Rat	Isoproterenol toxicity	Toxicological aggression	Severe structural modification by necrosis and fibrosis of myocardium	Less GEM animals and similar cost of equipment for cardiovascular physiology assessment than mouse
Rabbit	Pacing Tachycardia	Congestive failure by low output	Mimic myocardial alteration of human edematous chronic low output	Limited imaging technology due to paced heart rate (400 beats/min)
Rabbit	Balloon occlusion of circumflex branch of left coronary artery	Myocardial infarction	Artery occlusion by catheterization	Great skill and specific material
Dog	Pacing Tachycardia	Congestive failure by low output	Mimic myocardial remodeling, neurohumoral activation and subcellular dysfunction	No hypertrophy
Dog	Coronary microembolization	Contractile dysfunction and a profound perfusion-contraction mismatch	No surgery requirements	Microspheres are chemically inert. Extensive arterial pattern of heart. Time consuming
Pig	Pacing Tachycardia	Congestive failure by low output	Mimic myocardial remodeling, neurohumoral activation and subcellular dysfunction	No hypertrophy nor fibrosis
Pig	Coronary microembolization	Contractile dysfunction and a profound perfusion-contraction mismatch	No surgery requirements	Microsphere are chemically inert
Pig	Hibernating myocardium	Progressive reduction of ventricle perfusion	Mimic human disease condition	Surgical technical experience and skill. There is a myocardial recovery in chronic studies
Sheep	Pacing Tachycardia	Congestive failure by low output	Mimic myocardial remodeling, neurohumoral activation and subcellular dysfunction	No hypertrophy nor fibrosis
Sheep	Coronary microembolization	Contractile dysfunction and a profound perfusion-contraction mismatch	No surgery requirements and resemble human condition than dog	Zoonotic risk. Microspheres are chemically inert. Extensive arterial pattern of heart. Time consuming
**MYOCARDIAL INFARCTION**				
Mouse	Left coronary ligation (total occlusion or ischemia/reperfusion)	Myocardial infarction	Easy use of GEM animals, low cost of husbandry and feasible cardiovascular assessment. Suitability for follow-up and survival studies.	Great surgical skill and expensive technological requirements. Limited sample collection (animal size)
Rat	Left coronary ligation (total occlusion or ischemia/reperfusion)	Myocardial infarction	Surgical procedure easier than in mouse and more volume of samples. Lower cost than large animals. Suitability for follow-up and survival studies.	Less GEM animals and similar cost of equipment for cardiovascular physiology assessment than mouse
Rabbit	Left coronary ligation (total occlusion or ischemia/reperfusion)	Myocardial infarction	Surgical procedure easier than in rodents and more volume of samples Lower cost than large animals.	Thoracotomy surgery required
Dog	Left coronary ligation (total occlusion or ischemia/reperfusion)	Myocardial infarction	Surgical procedure easier than in rodents and more volume of samples Lower cost than large animals.	High death incidence by arrhythmias
Pig	Angioplasty balloon occlusion of the left anterior descending coronary	Myocardial infarction	Anatomy and pathology closed to human. Good suitability to undergo imaging techniques. No surgery requirements.	Require skills for coronary catheterization and surgical specific material
Zebrafish	Myocardial criolesion	Myocardial infarction	Heart remodeling and regenerative model	Far of mammals biology
**VASCULAR DISEASE**				
Mouse	APOE-deficiency and LDL Receptor deficiency	*Atherosclerosis*, Aortic root atherogenic lesions	Easy use of GEM animals, low cost of husbandry and feasible cardiovascular assessment. Great valuable data of molecular and cellular events.	Not mimic exactly the human chronic disease. The artery low size complicates the *in vivo* imaging acquisition
Rabbit	High-fat diet with/without balloon aortic injury	*Atherosclerosis*, Aortic arch and thoracic aorta lesions	Easy husbandry and feasible artery imaging acquisition.	Great skill for vessel damage, long term experimental induction of atherogenic lesions and no coronary affection
Rabbit	Watanabe WHHL (LDL Receptor deficiency)	*Atherosclerosis*, Aortic arch and thoracic aorta lesions	Easy husbandry and feasible artery imaging acquisition. Possible finding of coronary artery lesions. Not necessary high fat diet.	Unstable atherogenic plaque which could develop coronary occlusion and death
Pig	High-fat diet with/without angioplasty	*Atherosclerosis*, Aortic and coronary atherogenic lesions	Model closed to human disease	Long term experimental induction of atherogenic lesions. Skills for catheterism
**PULMONARY HYPERTENSION**				
Rat	Chronic Hypoxia	Increase in vascular tone	Repeatable maintained increase in pulmonary artery and RV pressure accompanied by RV remodeling	Minimal vascular remodeling. Suitable just for small animals
Rat	Chronic Hypoxia plus SU5416	Increase in vascular tone plus VEGFR-R blockade	Equal than chronic hypoxia more angiobliterative changes. More increase in RV pressure and more RV hypertrophy	Suitable just for small animals
Rat, dog, pig, sheep	Monocrotaline	Endothelial damage	Produces RV failure and vascular remodeling	No plexogenic arteriopathy
Dogs pig, sheep	Beads or clots injection	Decrease in total vessel area	Acute increase in pulmonary pressure RV remodeling	Decrease of the severity of vascular and RV changes with time. Hard to titrate the dose. High mortality in some reports
Pig, Rat	Aortocaval shunt	Increase in pulmonary artery flow	Resembles major features of human disease	Requires surgical skills. Complications related with surgery
Rodents, pig, sheep, dog	Vascular banding	Decrease in vascular compliance	Controllable and maintained increase in pulmonary artery pressure. RV remodeling	Requires surgical skills. Complications related with surgery

### Cardiac hypertrophy

Several strategies have been described to induce an adaptive response to pressure overload, sarcomeric mutations or pulmonary/artery hypertension, to mimic the hypertrophic transformation of the heart: (1) hypertrophic growth where load exceeds heart output, (2) study of compensatory events to normalize workload/mass ratio and cardiac output, and (3) HF because of ventricular dilatation (Meerson, 1961). Aortic and pulmonary artery stenosis are the most common methods for stressing the heart for a pressure overload (Tarnavski et al., 2004). The critical feature is to establish a normalized constriction of the artery to observe the increased difference between the left ventricular and aortic pressures or the difference between right ventricle (RV) and pulmonary artery. In non-rodent models, the normalization of the injury is fairly easy due to the feasibility of previous imaging studies. The measurement of the artery determines the precise restriction desired. A recent publication of RV failure in rabbits details the constriction induction of the pulmonary artery by the inflation of a 5-mm surgically implanted band controlled by the echocardiographic measurement of the right ventricular end-systolic pressure (McKellar et al., 2015). Imaging technologies are used for hypertrophic progress evaluation for the development of new therapies. However, the main disadvantage of these models is the period required to observe each stage of the cardiomyopathy, involving 2–3 months. On the other hand, the rodent models display an abrupt and acute hemodynamic instability. Heart morphological alterations are quickly acquired with a certain grade of variability depending of proportional reduction of the arterial lumen (−70%). The interest of the murine models is focused on the study of the compensatory events that occur after hypertrophic growth.

Other models that induce pressure overload without a required surgical expertise are: pulmonary hypertension (PH) by beads inoculation, genetic models of arterial hypertension and arrhythmogenic right ventricular cardiomyopathy. Murine genetic models and arrhythmogenic pathologies show a great advantage against other species. The spontaneous hypertension of rat (SH strains) or those induced in mutant mice are well characterized. However, the arrhythmogenic right ventricular cardiomyopathy of the Boxer and Hypertrophic Cardiomyopathy of MainCoon//Persian cats remain the best animal models for the study of both diseases regarding their similarities in genetic mutations etiologies and analogous mechanical-electrical dysfunctions (Kittleson et al., 1999; Basso et al., 2004; Palermo et al., 2011).

### Dilated cardiomyopathy

Animal models of dilated cardiomyopathy (DCM) should exhibit the structural and mechanical alterations observed in humans: LV dilatation, eccentric hypertrophy, wall thinning, reproducing cellular/molecular/neurohormonal features, depressed chamber output/flow, reduced ventricle contractility, and lusitropy, elevated filling pressure, and intolerance to stress situation due to a low functional reserve. Several strategies have been implemented (from rodent to large animal) to develop this disease phenotype.

The most used model is the myocardial ischemic injury, which can be performed with a permanent, temporary or progressive occlusion of the left coronary, just trying to mimic the atherosclerotic CAD. Depending on the animal species and surgical expertise, the technique can be performed in a closed or open-chest approach applying a surgical suture, beads microembolization, an intracoronary balloon-occlusion, or ameroid constriction. However, these experimental lesions are concentric contrary to the eccentric occlusion in human atherosclerosis (Bianco et al., 2009). Other strategies for the induction of congestive HF are: toxicological effect of doxorubicin or isoproterenol, pacing-induced tachycardia or genetic factors. In most of DCM animal models it is possible to observe a significant remodeling without severe clinical signs (Mann and Bristow, 2005). Asymptomatic LV dysfunction, reduced blood flow, elevated cardiac filling pressure, or other changes in hemodynamic could be absent while myocyte hypertrophy and fibrosis might be observed histologically. This is quite important to decide the experimental timing and the correlation with symptoms (Houser et al., 2012).

### Imaging in animals models of HF

Current non-invasive imaging techniques developed in research allows longitudinal evaluation of HF. Echocardiography remains as the gold standard for assessment of cardiovascular structure and function in rodents (Ram et al., 2011). However, CMR and PET-CT have gained importance in small animal research for further cardiac evaluation in the context of heart disease, especially evaluating anatomy and metabolism. In particular CMR is becoming an useful tool in both, systolic and diastolic HF studies in murine models (Loganathan et al., 2006; Chung et al., 2013; Constantinides, 2013). Literature on advance imaging protocols and cardiac echocardiography and MRI mouse models of HF is very extensive, therefore only those aspects helpful in the assessment of HF in mice are described.

Impaired systolic function is the main characteristic of HF with reduced EF animal models. Main cardiac findings are reduced LV EF, dilated or overload LV and coronary flow disturbs. Furthermore, assessment of LV regional wall motion abnormalities and calculation of wall motion score index offers to investigators an interesting tool to complement systolic dysfunction characterization and a measurement of heart damage extension, especially in ischemic injury.

Models of HF with preserved EF have predominantly LV diastolic filling alterations. Therefore, in these studies evaluation of mitral inflow pattern, pulmonary vein flow and left atrium dimensions could be essential for evaluating this condition. An important consideration to mention is that diastolic impairment does not noticeable affect cardiac output, so atrial diameter enlargement and pulmonary changes related to congestion could be useful to determine the develop of HF.

#### Mice transthoracic echocardiography

Two-dimensional (2D), motion-mode (M-mode) and color and pulse wave Doppler (CD and PW, respectively) are the basic techniques used in research (Ram et al., 2011; Chen et al., 2012; Moran et al., 2013). Parasternal long axis view (PLAX) and parasternal short axis views (PSAX, at basal, medium, and apical) in 2D and M-mode allows entirely LV visualization, therefore LV function, regional wall motion as well as chamber and wall dimensions can be accurately determined (Zhang et al., 2007; Fayssoil and Tournoux, 2013). Apical four-chamber view is used to visualize both ventricles and as ultrasounds can be aligned with blood flow through mitral and PW Doppler is set in this plane to study mitral inflow pattern. Additional views or PLAX-angled views have also been described in mice to study coronary, pulmonary vein and pulmonary artery flows, which can be useful in the context of diastolic and systolic dysfunction (Wu et al., 2012; Cheng et al., 2014).

Conventional echocardiographic measures lack sensitivity for capturing subtle variations in global ventricular performance (Bauer et al., 2011). In this regard, novel echocardiographic techniques based on tracking tissue motion have emerged for clinical use and animal cardiac research to assess strain. These may provide quantitatively evaluation of myocardial function and early detection of ventricular performance alterations. Doppler tissue imaging (DTI) and speckle-tracking imaging (STI) based on strain analysis are the main echocardiographic tools described in human and small animal models in this regard, although current applications remain limited (Mor-Avi et al., 2011). In DTI, the same Doppler principles are used to identify signals of myocardial tissue motion. Peak myocardial velocities of early and late diastolic and systolic waves can be measured (also in mice) in the anterior/posterior LV wall for the radial component, and in the lateral free LV wall for the circumferential component on the PSAX view (Ho and Solomon, 2006; Mor-Avi et al., 2011; Ferferieva et al., 2013). STI based of strain analysis detects the gray scale “speckles” within the tissue on 2D ultrasound imaging and its movement during cardiac cycle and has been recently used for the estimation of the radial and circumferential strain in small animal models (Mor-Avi et al., 2011; Ferferieva et al., 2013).

In mice, assessment of deformation parameters through DTI or STI based on strain analysis correlated well with invasive hemodynamic measure of myocardial contractility and both techniques could be equally acceptable for assessing LV function (Ferferieva et al., 2013). However, these techniques are not yet routinely established in the echocardiography protocols of cardiac disease. The use of DTI and STI based on strain analysis to provide good cardiovascular phenotype in mice are of great interest but improvement of the analysis should be develop to make these new tools robust, reproducible and more user-friendly (Fayssoil and Tournoux, 2013).

In HF with reduced EF models, typically derived from systolic alterations, LV EF is the major parameter to determine this condition and can be determined from both, 2D and M-mode PLAX views. It is important to mention that in ischemic injury, M-mode echography cannot accurately estimate LV EF because the ventricle shape is abnormal, and geometric models and algorithms assumptions cannot be assumed. In these cases, EF is better estimated using the area-length formula in an 2D PLAX view; if additional 2D PSAX has been taken at basal, medium and apical, Simpson formula to calculate LV EF and regional wall motion can also be performed. LV dilatation or overload can be demonstrated obtained the end-diastolic volume of the LV, usually from 2D PLAX. Evaluation of coronary flow in murine models of HF with reduced EF could be essential in some cases as coronary disturbance could lead to abnormal myocardial perfusion and therefore could compromise systolic function. Hyperemic peak diastolic velocity and coronary flow reverse are the principal changes in coronary flow during ischemic injury and reduced EF (Wu et al., 2012).

Echocardiography evaluation of HF with preserved EF in mice, normally derived from diastolic alterations, can be challenging as small heart size and rapid ventricular rates make evaluation of diastolic function difficult (Chung et al., 2013). Variation in the mitral flow is the most consistent finding in these studies. Mitral inflow pattern can be achieve in the apical view, and common parameters altered are E wave-A wave ratio, isovolumetric relaxation time, E wave deceleration time and A wave time (Wichi et al., 2007; Ram et al., 2011; Fayssoil and Tournoux, 2013). Pulmonary vein flow changes are commonly used in human echography and could be a future marker of diastolic dysfunction in rodents but no consistent changes have been described. Pulmonary veins in these animals differ from human as venous confluence with a single orifice enters the left atrium in mice, thus, altering the pressures and, therefore, the wave patterns (Yuan et al., 2010).

Left atrial dilatation measured form 2D and M-mode long-axis has been described as a parameter indicating pulmonary congestion (Finsen et al., 2005), therefore, it could be used in both, reduced and preserved EF models, but could be especially useful in preserved EF where reduced cardiac output cannot be assessed. Pulmonary artery flow varies depending on lung abnormalities and mice have similar pattern to that observed in humans (Thibault et al., 2010). In both, HF with reduced or preserved EF, if there is a maintained pulmonary congestion, high pulmonary pressures can be achieved and typical pattern of hypertension can be visualized. Acceleration and total ejection time ratio is reduced in accordance with increase in pulmonary pressure in hypertension murine models (Thibault et al., 2010), whereas in some models of HF such as ischemic injury, the changes in arterial flow have not been very consistent with the presence or absence of pulmonary congestion (Finsen et al., 2005).

#### Mice cardiac MRI

**Figure 4:**
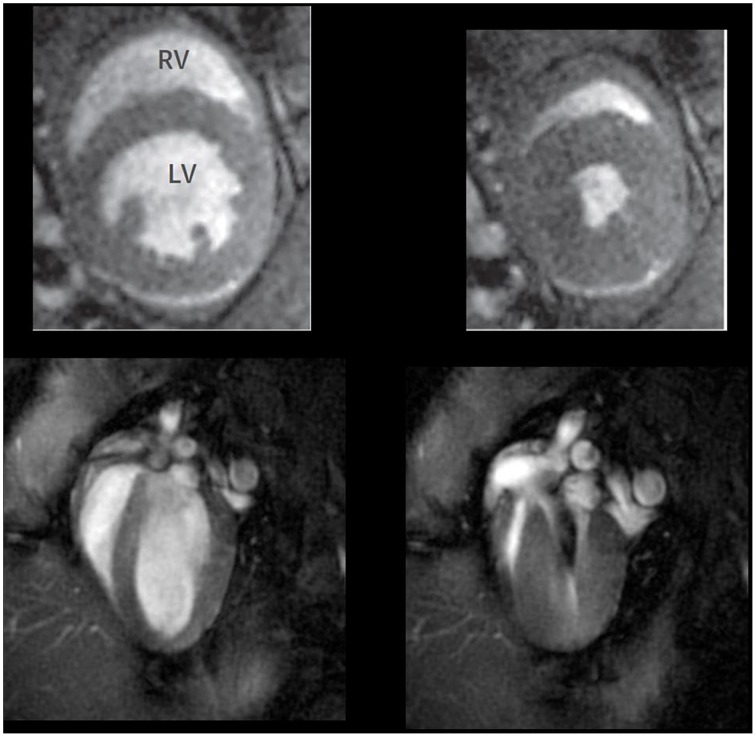
**Representative short axis (upper panel) and long axis (low panels) cardiac magnetic resonance images taken at the end of diastole (left panels) and systole (right panels) in WT mice**. Adapted from Figure 3 in Cruz et al. (2015).

*In vivo* MRI provides excellent views of cardiac structures and allows high quality spatial resolution of the heart, and recently has emerged as an accurately instrument for functional cardiac evaluation as high temporal resolutions are also achieved (Yue et al., 2007; Figure 4). Cardiac gating is essential to reduce the level of motion artifacts and therefore, to obtain images of sufficient quality (Cassidy et al., 2004). Assessment of cardiac anatomy, regional wall motion, myocardial perfusion, myocardial viability plus cardiac chamber quantification, and cardiac function are applications described in mice (Pohlmann et al., 2011). Conventional views in mice are similar to those used in human cardiac MRI. Long-axis, two and four-chamber views as well as a multi-slice short-axis view from base to apex are commonly used in bright-blood and black-blood spin-echo.

CMR in HF with reduced EF models is becoming progressively more used as it allows precisely data of LV volumes with no need of making geometric assumptions regarding ventricular shape, which is especially important in ischemic injury models. In these models, MRI also allows measuring the total infarcted area as the scar can be well visualized. Besides, the high anatomical resolution and improvements achieved in temporal resolution make MRI a powerful tool to assess cardiac systolic dysfunction and wall motion, not only in LV but also in the RV. Normally, LV and RV EF as well as volumes are calculated after obtaining planimetry of each short-axis slice in end-systole and end-diastole.

As it has been describe previously in this report, non-invasively imaging evaluation of diastolic dysfunction in rodents is limited because of the short diastolic period. As most of the HF with preserved EF models are characterized by diffuse myocardial fibrosis and structural alterations, MRI has become an important imaging technique to describe cardiac mass, tissue changes, and interstitial fibrosis through special analysis as pixel intensity values (Loganathan et al., 2006). Furthermore, evaluation of LV filling rate calculated from slopes of the volume-time curves can also provide relaxation information useful for determining diastolic dysfunction in these models (Yu et al., 2007). Finally, CMR can be simultaneously used to assess the progression of ventricular dysfunction with lung congestion. This non-invasive technology is especially applicable to serial studies and in drug discovery programs exploring the use of novel pharmacological or molecular agents for treatment of heart failure and/or pulmonary congestion (Alsaid et al., 2012).

### New challenge in heart failure imaging

**Figure 5:**
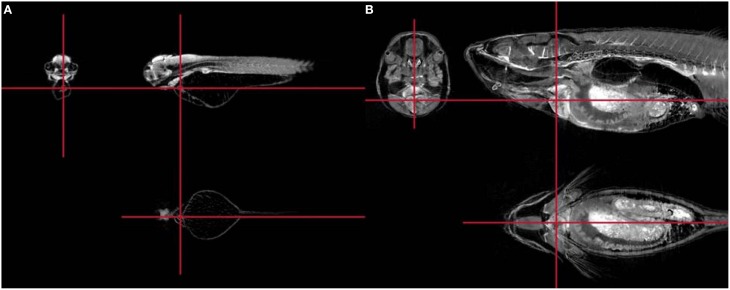
**Representative high resolution MRI sections to visualize the normal heart structure of zebrafish embryo (A) and an adult fish (B)**. From Bryson-Richardson et al. (2007) Figure 1 with original publisher's permission (Bio Med Central).

One of the ultimate goals of HF treatment is aimed to cardiac repair by the regeneration of the myocardium after injury. The mammalian heart is one of the least regenerative organs in the body, although postnatal hearts undergo some degree of cardiomyocyte renewal during normal aging and disease. Together with mouse, zebrafish has proven to be a particularly useful model, given its amenability to genetic manipulation and its tissue regenerative capacity (Poss et al., 2002). Zebrafish models are directed to observe the cardiomyocyte proliferation after heart ablation or myocardium crioinjury. Significantly, the principal limitations of this model is the handling and HF procedure performing in an aquatic species, the anatomical differences of cardiovascular system with mammals and the setting up of imaging techniques for hemodynamic measurements (González-Rosa et al., 2014). However, the interspecies jump is too high to conclude the evidence acquired in zebrafish cardiovascular studies for human or mammal cardiac diseases. For that purpose, heart regeneration models have been established in mouse neonates. Obviously, the cardiac repair differs from zebrafish, but the cardiac insult is quite similar. Crioinjury infarct and apex ablation are basically the preference model in this field. Surgical expertise and special handling are crucial for the success of the technique. The main point in the surgical part is the promptness to open the chest and damage the myocardium. The anesthetic regimen is based on the immobility by cold, which should be recovered as soon as possible. Later, the most important *tour de force* is the reinstatement to the mother due to the easy induction to cannibalism by the extremely sensitive olfactory and visual sense (Laflamme and Murry, 2011). While a number of imaging methods and applications have been shown mainly using optical microscopy, MRI can be adapted and can provide additional *in vivo* information (Figure 5).

## Atherosclerosis and vascular lesion characterization

Atherosclerosis is a chronic inflammatory disease, affecting the medium and large arteries, characterized by the progressive accumulation of lipid deposits and different cell types in the subendothelial space (mainly immune cells and vascular smooth muscle cells) to form the atherosclerotic plaques. The major clinical manifestations of atherosclerosis are CAD, leading to acute MI (see Section Imaging in Myocardial Infarction and Coronary Artery Disease); cerebrovascular disease, leading to stroke; and peripheral arterial disease, leading to ischemic limbs and viscera (Libby, 2002). Traditionally, diagnosis of atherosclerosis was only possible at advanced stages of disease, either by directly revealing the narrowing of the arterial lumen (stenosis) or by evaluating the effect of arterial stenosis on organ perfusion. However, it is now well recognized that the atherosclerotic plaques responsible for thrombus formation are not necessarily those that impinge most on the lumen of the vessel. New imaging approaches allow the assessment not only of the morphology of blood vessels but also of the composition of the vessel walls, enabling atherosclerosis-associated abnormalities in the arteries to be observed, down to the cellular and molecular level in some cases. Some of these approaches are now in clinical use or are being tested in clinical trials, whereas others are better suited for basic and translational research (Sanz and Fayad, 2008; Owen et al., 2011).

### Animal models of atherosclerosis

Experimental models of atherosclerosis are required to understand the natural history of the disease and the factors leading to plaque progression and rupture. Thus, ideally, animal models of atherosclerosis should present a progressive development of arterial lesions throughout life, from initial fatty streak to advanced complicated lesions (ulceration, thrombus, etc…), with shared histological features and clinical events (MI, stroke) similar to humans (Russell and Proctor, 2006; Fuster et al., 2012).

Animal models of atherosclerosis started emerging in the literature at the beginning of the twentieth century with rabbits being the first animal species to be described (Ignatowski, 1908). Since then, a wide variety of animal models, including mice, rats, guinea pigs, hamsters, avian, swine, and non-human primates, have provided valuable information about diagnostic and therapeutic strategies for atherosclerosis (Xiangdong et al., 2011; Fuster et al., 2012). Currently, mice, rabbits, and pigs dominate this field of research. Susceptibility to atherosclerosis varies not only between animal species but also between genetic strains within the same species.

The mouse is the most frequently used animal species in atherosclerosis research. Some of the reasons include low costs, availability, rapid development of plaque, well-known genome, possibility of genetic manipulations, and easy usability. But mice are highly resistant to atherosclerosis as compared to humans, due in part to a markedly different lipid profile. Therefore, genetic variants that do develop the disease have been created either by spontaneous mutations or by gene manipulation methods. The majority of mouse models are based on disturbance of lipid metabolism through genetic manipulation of the C57BL/6 strain. Among those, the apolipoprotein E-deficient mouse (apoE-KO) is the most distributed worldwide. Arterial lesions observed in apoE-KO are relatively similar to lesions found in humans: from foam cell-rich fatty streaks to atherosclerotic lesions with large necrotic core, and fibro-fatty nodules. In mice, the most advanced lesions have been described to occur in the aortic root, aortic arch, and innominate artery (brachiocephalic trunk), whereas in humans the lesions are more frequent in the coronary arteries, carotids, and peripheral vessels, such as the iliac artery (Nakashima et al., 1994). In addition, most murine models do not manifest the unstable atherosclerotic plaque with overlying thrombosis, the lesion most often associated with clinically significant acute cardiovascular episodes. This is due to a spontaneous high fibrinolytic activity in the mouse (Zhu et al., 1999). Moreover, the size of the arteries makes it extremely difficult to image with sufficient resolution in clinical scanners used for humans, precluding the efficient translation of techniques from animal model to human scanning.

When the size becomes more important, small animal models need to be complemented by larger animal models in which vessel characteristics are more similar to human arteries. Rabbits develop atherosclerotic lesions on a high cholesterol diet, and have been used extensively in atherosclerosis research (Yanni, 2004). The most widely used, the New Zealand white rabbit, develops fatty streaks and intimal thickening when fed a high cholesterol diet, with most of the lipids localized within the profuse macrophage-derived foam cells. Lesions are preferentially localized in the aortic arch and the thoracic aorta. More advanced lesions can be induced by performing a balloon injury of the aorta or the carotid artery and further fed with high fat diet. This combined protocol accelerates the formation of atherosclerotic lesions and produces plaques that exhibit a lipid core surrounded by a fibrous cap due to increased proliferation of vascular smooth muscle cells, thus resembling more closely human advanced plaques (Badimon, 2001). Like mice, some rabbit strains carry genetic mutations that lead to hyperlipidemia and atherosclerosis, with the Watanabe heritable hyperlipidemic rabbit being the most commonly used; these animals spontaneously develop lesions due to an inactivating mutation in the gene encoding the LDL receptor (Watanabe, 1980). In these animals the atherosclerosis progresses with age even with a cholesterol-free diet.

One major advantage of the rabbit model is the relatively small animal size, which makes it easy to care, handle and feed, and therefore inexpensive, but large enough to monitor physiological changes and use clinical scanners. Rabbits are favored when imaging arteries; the diameter of the rabbit aorta is 2–4 mm, comparable to the size of human coronary arteries. Imaging techniques such as ultrasound or MRI can be effectively applied to determine the plaque composition and its vulnerability (Helft et al., 2002; Wetterholm et al., 2007; Phinikaridou et al., 2010). The major disadvantage of this model is the relatively slow progression of the pathological condition and the required length of the studies.

However, when trying to obtain close-to-clinical models, pigs and minipigs seem to be the most representative ones. Pigs have a highly comparable anatomy and physiology of the coronary system with humans (White et al., 1986), as well as a very similar lipoprotein profile and metabolism (Dixon et al., 1999). Unlike mice and rabbits, pigs develop spontaneous atherosclerosis during aging, and the development of plaques can be induced by diet, hyperglycemia and by introducing vascular injury, usually by angioplasty. In addition, with use of toxin-mediated pancreatic damage and a high fat diet, human diabetes mellitus-like metabolic alterations will develop, followed by coronary lesions resembling the human condition closely, with even some characteristics of vulnerable plaque (Gerrity et al., 2001). Also, porcine models of familial hypercholesterolemia based on delayed LDL clearance have been shown to develop complex atherosclerotic lesions. The lesions are preferentially localized in the aorta, coronary and iliac arteries, and are characterized by necrotic cores, calcification, neovascularization and intraplaque hemorrhage, closely mimicking advanced human plaques (Prescott et al., 1991). However, the size and composition of the LDL differ in the high-fat diet fed pigs and the familial hypercholesterolemic pigs (Checovich et al., 1988). Although pigs are an excellent model for studying the basic mechanisms, pathophysiology, and progression of atherosclerosis, their high cost of maintenance and longer diet-induction time limit the use of pigs in research of atherosclerosis on a large scale.

### The imaging modality of choice for atherosclerosis assessment

Different non-invasive imaging modalities can provide information about plaque anatomy and composition. Nonetheless, post-mortem histological analysis of atherosclerotic lesions in experimental animal models (and human autopsies) is fundamental to validate the model and the technique and remains the gold standard.

Ultrasound imaging can be used for plaque detection and analysis in animal models of atherosclerosis (e.g., rabbit, monkey), typically by measuring carotid or aortic wall thickness using high-frequency ultrasound transducers (Zeng et al., 2015). Determination of plaque composition with ultrasound is based on tissue echogenicity: hypoechoic heterogeneous plaque is associated with both intraplaque hemorrhage and lipids, whereas hyperechoic homogeneous plaque is mostly fibrous; however, precise plaque characterization (lipid core, intraplaque hemorrhage, or fibrous cap rupture) is not possible using non-invasive ultrasound measurements and image acquisition is complex and operator dependent. Notably, recent advances with contrast-enhanced ultrasound based on microbubbles have allowed detection of plaque neovascularization and disease progression monitoring (Giannarelli et al., 2010; Nitta-Seko et al., 2010; Tian et al., 2013).

CT is also well suited for studying atherosclerosis non-invasively in all vascular regions, although it requires the use of ionizing radiation and contrast agents. The main advantages of CT include its high spatial resolution and the short scan times, but its major disadvantage for atherosclerotic plaque characterization is the limited discrimination of soft tissue. Although vascular calcification is the main target of this imaging modality, CT can also provide reasonably accurate quantification of plaque size and crude characterization of plaque composition on the basis of lipid-rich tissue attenuating X-rays to a smaller extent than fibrous tissue (Viles-Gonzalez et al., 2004; Cordeiro and Lima, 2006). The development of micro- and nano-CT has allowed higher spatial resolution, enabling *in vivo* and *ex vivo* studies in small animal models (Awan et al., 2011). CT imaging can also be merged with positron emission tomographic (PET) modalities, most prominently with ^18^F-fluorodeoxyglucose (^18^F-FDG), to study the metabolic state of the vascular lesion in addition to its anatomical features (Vucic et al., 2011).

**Figure 6:**
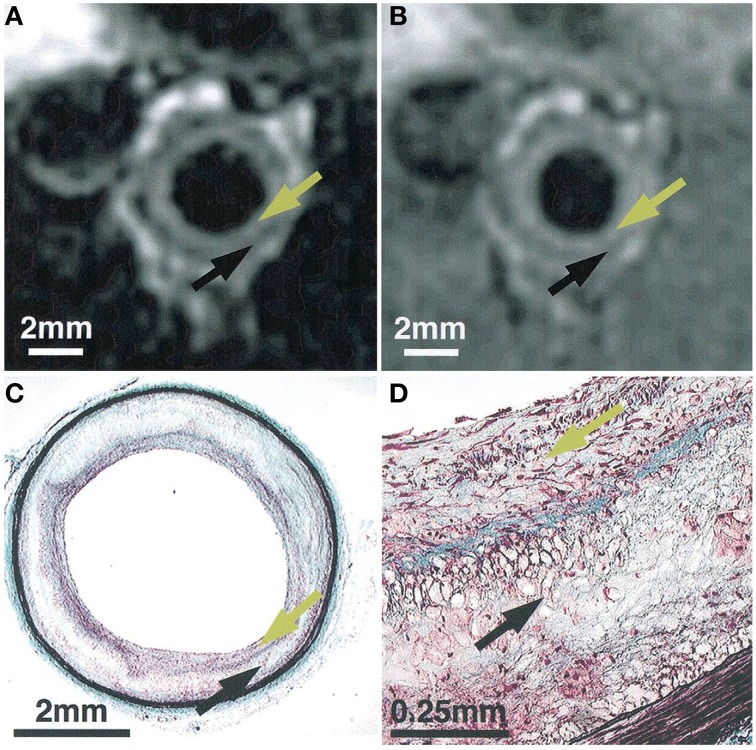
**T2-Weighted (T2W) (A) and Proton Density (PD) *in vivo* MR image (B) of a rabbit abdominal aorta with advanced atherosclerosis**. **(C)** Corresponding histopathological section with Masson's trichrome elastin stain showing fibrotic and lipid components and magnification **(D)** showing the foam cell-rich lipid regions and the fibrotic cap. Taken from Helft et al. (2001) with permission.

MRI can provide information on both plaque burden and composition in multiple arterial territories. Compared with other imaging modalities, MRI appears to have the greatest potential for plaque characterization. Based on differences in the biophysical and biochemical properties of the different components of the plaque, it is possible to determine the anatomy and composition of the lesions (lipid-rich necrotic core, fibrous cap, calcification, fibrosis) by combining multiple contrast sequences (T1, T2, and proton-density weightings; Corti and Fuster, 2011). Vulnerability criteria such as ulceration, cap rupture and intraplaque hemorrhage are also easily identifiable. The main advantages of MRI include its high spatial resolution, great soft-tissue contrast, lack of radiation exposure and good clinical translatability. Several studies have validated the feasibility of high resolution MRI to quantify atherosclerosis progression/regression and to determine composition of atherosclerotic lesions in animal models, including mouse (Fayad et al., 1998), rabbit (Helft et al., 2001; Ibanez et al., 2012), and pig (Worthley et al., 2000). Figure 6 shows an example of *in vivo* MRI plaque characterization of rabbit abdominal aorta. Furthermore, use of contrast agents such as gadolinium (Koktzoglou et al., 2006) or ultrasmall paramagnetic iron-oxide particles (Millon et al., 2013) and the possibility of combination with PET (Davies et al., 2005; Millon et al., 2013) can provide information on the inflammatory activity and the presence of macrophages in the plaque. Thus, MRI is an excellent imaging modality to monitor atherosclerotic plaque formation and destabilization in preclinical animal models. Multimodal molecular imaging approaches such as PET/CT, PET/MRI, MRI-optical, and PET-optical focuses in identifying cellular and molecular targets (e.g., dysfunctional endothelium, mineral deposition, enzymatic activity, inflammation, neovascularization, thrombosis, extracellular matrix production, etc…), and is playing a role in understanding these key biological processes of atherogenesis *in vivo*. The use of coordination chemistry (including various chelates depending on the final application) or colloidal chemistry including metallic nanoparticles or nanosystems such as biocompatible polymers or liposomes, modified or coated with peptides, proteins or antibodies for increasing *in vivo* specificity and target specific molecules. One very recurrent demonstration is the imaging of the vascular cell adhesion molecule (VCAM-1) for what different radiolabeled ligands (Nahrendorf et al., 2009) or nanoparticles functionalized with different molecules have been tested (Kelly et al., 2005; Nahrendorf et al., 2006; Broisat et al., 2012; Michalska et al., 2012). In the road for translation, it is especially relevant the role of different animal models. There are many examples in the bibliography and excellent reviews are continuously covering these new aspects (Nahrendorf et al., 2009; Mulder et al., 2014). These advances are also relevant in other cardiovascular conditions, but for the sake of simplicity we are not covering them.

In addition to the aforementioned non-invasive techniques, optical coherence tomography (OCT), a novel invasive catheter-based technology, has gained much interest as a preclinical tool for plaque characterization. OCT is analogous to ultrasound, measuring the back-reflection of infrared light rather than sound. OCT generates high-resolution (5–20 μm) three-dimensional images with high data acquisition rates and small and inexpensive guidewires/catheters. Its main drawback is the limited tissue penetration (1–3 mm; Stamper et al., 2006).

## Animal models for pulmonary hypertension image studies

Animal models are indispensable for the advance of imaging in PH. In this disease imaging tools have helped to the mechanistic understanding of PH evaluating pulmonary vasculature and RV function and allowing identification of innovative biomarkers. It is important to recognize that PH accomplish different conditions that finally share a common finding, the chronic increase in pulmonary artery pressure. The conditions that lead to the development of PH are grouped according to the Nice Classification (Simonneau et al., 2013). At the time we need to choose an animal model, this should fit: (a) the primary change that is related with the specific PH that we want to study and (b) the functional and structural changes that occurs in cardiovascular system as consequence of the disease.

Special attention deserves the RV as this is the major determinant of functional state and prognosis in PH (Ryan and Archer, 2014). Usually when RV failure is late diagnosed, patient mortality in a short term and worsening quality of life are increased. In this way, implementation of imaging tools in animal models can be used for detection of early changes in RV and pulmonary vessel function that could be modified preventing worsening and failure.

**Figure 7:**
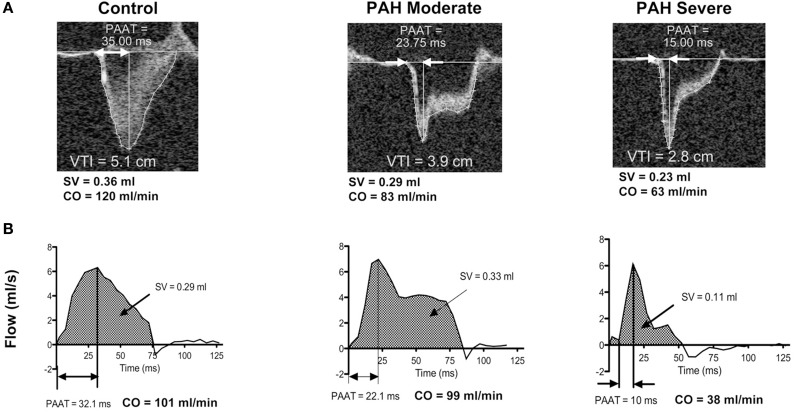
**Representative echocardiography (A), MRI (B) pulmonary artery flow in controls and experimental rats with moderate and severe pulmonary hypertension**. VTI, velocity time integral; PAAT, pulmonary artery acceleration time; SV, stroke volume; CO, cardiac output. Modified from Urboniene et al. (2010) Figure 2 with permission.

Echocardiography is probably the most extendedly used imaging tool in PH in both, clinical practice and animal studies. An estimation of the pulmonary artery pressure can be done using a modified Bernoulli equation. Doppler echocardiography provides a non-invasive assessment of the RV structure and function, and it is used to monitor progression and response to therapy. The interval between the onset of flow and peak flow of the pulmonary artery (pulmonary artery acceleration time) is inversely related with pulmonary artery pressure and can be used to estimate the severity of PH (Figure 7).

MRI is becoming the gold standard in the study of patients with PH and also its application in animal studies is increasing. MRI facilitates a well-detailed anatomical description of RV. Using MRI important advances have been done in the description of flow in 2D and 3D (4D) bringing new insights related with functional hemodynamic changes during PH. MRI allows the simultaneous measurement of pulmonary artery area and flow velocity allowing complex hemodynamic assessment. This assessment includes the calculus of wave speed and the evaluation of wave reflection phenomena. Also, PA distensibility can be estimated by area changes in PA.

CT can be a diagnostic and also a complimentary tool in PH. It is nowadays the reference tool for diagnosis of pulmonary embolism, which is involved in the etiology of chronic thromboembolic PH. CT provides unbeaten structural information of the lungs which is essential to reveal the direct relation between lung parenchyma and pulmonary vessels.

PET is less used in clinical practice and is not a routine tool for the evaluation of PH patients. However, new mechanistic insides are described due to its ability of tracking complex process such as cellular proliferation, metabolic pathways, inflammation, and ventilation/perfusion distribution. These processes can be critical in the understanding of PH physiopathology and can also be used in the near future for patient follow-up and therapeutic targeting. All this has made PET a promising field for new animal and clinical studies.

No animal model is perfect in any of the PH types. This is due to the complex changes related with vessel remodeling, which involve different molecular and cellular pathways and depend on multiple variables. Models that combine multiple insults yield more severe PH with better hemodynamic and histological fidelity to human pathology. However, animal models can represent specific changes that can be studied. Given this, we can describe the different animal models used in PH as follows:

- An increase in the pulmonary vasomotor tone caused by hypoxia. This model resembles pulmonary arterial hypertension (Group 1 of the Nice classification). Hypoxia can be created in a normobaric or hypobaric environment. This model is used in small animal, as the animal should be enclosed in the hypoxic environment. Rats exposed to this model show a decrease in blood flow velocity measured by dynamic MRI (de La Roque et al., 2011). Novel experimental treatments for PH have been tested using this model. For example, treatment with dehydroepiandrosterone improves the right ventricular systolic and diastolic functions assessed by Doppler echocardiography (Dumas de La Roque et al., 2012).

Although causing an elevation in pulmonary artery pressure, chronic hypoxia is associated with minimal vascular remodeling, which limits its use (Ciuclan et al., 2011). More recently, vascular endothelial growth factor receptor (VEGF-R) blockade with the tyrosine kinase inhibitor SU5416 have been introduced and the SU5416 plus chronic hypoxia model is now being extensively used (de Raaf et al., 2014). This model features angio-obliterative PH resembling human pulmonary arterial hypertension and also increases RV pressure and RV hypertrophy comparing to mice just submitted to chronic hypoxia (Ciuclan et al., 2011). Dehydroepiandrosterone was also tested in this model. Echocardiography data showed that this drug significantly reduced RV internal diameter during diastole and restored the normal position and orientation of the interventricular septum, which had been otherwise flattened (Alzoubi et al., 2013).

- Endothelial damage produced by monocrotaline. This model also resembles Group 1 of the Nice Classification. Monocrotaline elicits pulmonary arterial endothelial cells dysfunction on multiple levels but is characterized by pulmonary artery arterial medial hypertrophy and not by endothelial cell-mediated angioobliteration (the last is present in the human disease and in the SU5416/hypoxia model). Monocrotaline has been well described to produce RV failure, however is criticized because it does not produce plexogenic arteriopathy, but produces left and RV myocarditis (Gomez-Arroyo et al., 2012). Using FDG PET authors have shown an increase in glucose uptake during compensated RV hypertrophy, which was reversed during decompensated RV hypertrophy (Sutendra et al., 2013). This behavior was paralleled by activated hypoxia-inducible factor 1 α (HIF1α) and angiogenesis evaluated by means of lectin imaging *in vivo* (Sutendra et al., 2013). Several proposed treatments have been evaluated in this model. Using echocardiography, sildenafil, pimobendam (a calcium sensitizer and phosphodiesterase inhibitor) and nicorandil (a vasodilator) were tested. The 2D mode in echocardiography and tissue Doppler imaging data indicated improved cardiac morphology and function in all treatment groups (Nakata et al., 2015). Authors showed that Carvedilol improves fibrosis and hemodynamic behavior despite persistent RV increased afterload. Carvedilol improved tricuspid annular displacement and global LV radial and RV longitudinal strain (Okumura et al., 2015). Other investigators tested Macitentan therapy showing an increase in pulmonary artery acceleration time and a decreased deceleration time and decreased RV wall thickness with this therapy (Temple et al., 2014).
- Decrease in the total vessel area produced by clots or beads injection. These models resemble chronic thromboembolic PH (Group 4 of the Nice classification). An increase in pulmonary artery pressure and resistance as well as signs of RV remodeling have been described after repeated injections of beads. Different protocols of injection has been tested to find an important increase in pulmonary artery pressure and resistance without causing death of the animal. Using microbeads injection in dogs authors have demonstrated that both magnitude and flow-sensitive data from a single 4D flow CMR acquisition permit simultaneous quantification of cardiac function and cardiopulmonary hemodynamic parameters important in the assessment of PH (Roldán-Alzate et al., 2014). The same authors demonstrated the benefit of using the same sequence for measuring cardiac chamber volumes and flow resulting in an overall shortened examination acquisition time (Roldán-Alzate et al., 2014).
- Increase in the pulmonary artery flow produced by shunt. The mechanisms of PH with left-to-right shunt are different from those of hypoxia and inflammation. Possible inciting factors of the former are mechanical stretch and shear stress. MRI was evaluated in rats with aortocaval shunt to reliably produce right ventricular volume overload and secondary PH. Due to a combination of left ventricular dysfunction and pulmonary overflow, the PH produced show features similar to those found in patients with chronic atrial-level shunt. The RV end-diastolic area and the thickness of the RV free wall increased significantly and a mesosystolic notch was demonstrated in the Doppler wave profile of the pulmonary flow after 20 weeks of shunt (Linardi et al., 2014).
- Pulmonary vessels (artery or vein) decrease in compliance and increase in resistance produced by banding. These models resemble PH due to left heart disease (Group 2 of the Nice Classification). Selecting artery or vein a model of pre or post-capillary PH can be developed. A porcine model of postcapillary PH by non-restrictive banding of the confluent of both inferior pulmonary veins was developed and evaluated (Pereda et al., 2014). All banded animals developed PH. Ventricular remodeling produced progressive increase in end diastolic and systolic volumes of the RV, an increase in its mass, and an EF decrease. Also this model consistently reproduced most pathology changes usually seen on pulmonary arterial circulation, including intimal fibrosis (Pereda et al., 2014). In a precapillary PH model by pulmonary artery banding, early compensatory mechanisms of the RA and RV response to RV pressure overload were investigated (Voeller et al., 2011). In this study, MRI with tissue tagging was used to measure circumferential and minimum principle strain. The resulting data revealed that early RV pressure overload, without chamber enlargement, has a measurable effect on RA function and RV strain patterns when overload is severe. With a 2.5-fold rise in RV afterload, RV filling became more dependent on RA conduit than reservoir function, which likely reflects loss of RV diastolic compliance and consequent stiffening of the RA and RV walls (Voeller et al., 2011).
- In some studies, authors have combined different models of PH. For example, using the monocrotaline plus pulmonary artery banding, the inhibition of pyruvate dehydrogenase kinase by dichloroacetate on RV hypertrophy was evaluated (Piao et al., 2010). The rationale for combining two models was that in the monocrotaline model, there is an intrinsic coupling between severity of RV hypertrophy and severity of the pulmonary vascular disease, while pulmonary artery banding results in RV hypertrophy secondary to pure pressure overload. The study was performed by echocardiography and PET. FDG uptake in RV and the RV/LV FDG was increased in monocrotaline and pulmonary artery banded rats. Dichloroacetate tended to reduce FDG uptake ratio and also improved the pulmonary artery acceleration time (Piao et al., 2010).

A recent report includes three different models (one acute and two chronic) of PH and one vasodilator testing to validate a CMR estimation of pulmonary vascular resistance (García-Álvarez et al., 2013). Acute and chronic beads embolization and pulmonary vein banding were used as models of PH. They showed that changes in pulmonary artery velocity inversely correlate with pulmonary vascular resistance and that MRI can be used to track acute and chronic in PVR (García-Álvarez et al., 2013).

## Cardiovascular imaging for translational research

Nowadays, we have the possibility of using in animal research the same cardiovascular imaging technology used for human diagnosis to establish the severity and prognosis of diseases. Imaging systems and methods previously described provide a common link between humans and experimental animal models for identifying common phenotypes of cardiovascular diseases in their functional and morphological aspects. Tissue characterization by MRI in the evaluation of necrosis, fibrosis, fat, perfusion, flow, and myocardial edema and in the evaluation of cardiovascular pathologies is one of the vanguard tools for clinical and translational research. Nuclear medicine based techniques for molecular imaging using PET or SPECT for myocardial metabolic and perfusion characterization have been used for years. PET is currently used for human atherosclerosis and FDG PET has recently gained a role in the assessment of patients with PH. Due to the high sensitivity of these nuclear medicine based protocols, applications are surely in a prominent position to investigate novel metabolic and cellular targets in animal models with potential human translation. Similar or alternative molecular procedures for MRI are based in Gadolinium, Fluorine, iron oxide nanoparticles, and recently in hyperpolarization procedures (Schroeder et al., 2008; Bhattacharya et al., 2009; Rider and Tyler, 2013). Despite the many examples targeting multiple molecular and cellular pathways in animal models, the reduced sensitivity of MRI probably hampers its further development on translational research. However, cardiovascular hybrid imaging including MRI technology are leading tools for new translational research, since they provide simultaneous or sequential acquisitions covering molecular, functional, cellular and tissue characterization of different cardiovascular disease phenotypes.

## Conclusions

Imaging technologies for the study of cardiovascular diseases are in constant progress and their relevance in the guidance of patients will probably expand in the next years. Specific structural, functional and molecular changes will be surely be observed by imaging technologies. In this process, the discovery of new pathophysiological pathways will bring new approaches for both diagnostic and therapeutics targets. This growth will also generate new hypotheses and original research lines will consequently emerge. During this phase, any innovative imaging technology will require to be tested in specific disease mimicking malignant conditions and new imaging tracers to study particular molecular and physiological changes will also be evaluated using imaging methodologies. All these examples will always require the optimization of animal models trying to find a better approach to the human disease.

## Author contributions

AS, LF, MV, BL, SE, JM, RM, JJ, and JR participated in the conception, design and draft of this review and agree to be accountable for all aspects of the work in ensuring that questions related to the accuracy or integrity of any part of the work are appropriately investigated and resolved.

## Funding

AS and SE are M+Visión COFUND Advanced Fellows and have received funding from Consejería de Educación, Juventud y Deporte of Comunidad de Madrid and the People Programme (Marie Curie Actions) of the European Union's Seventh Framework Programme (FP7/2007-2013) under REA grant agreement n° 291820.

### Conflict of interest statement

The authors declare that the research was conducted in the absence of any commercial or financial relationships that could be construed as a potential conflict of interest.

## References

[B1] Abarbanell A. M. Herrmann J. L. Weil B. R. Wang Y. Tan J. Moberly S. P.. (2010). Animal models of myocardial and vascular injury. J. Surg. Res. 162, 239–249. 10.1016/j.jss.2009.06.021 20053409

[B2] Adams M. C. Turkington T. G. Wilson J. M. Wong T. Z. (2010). A systematic review of the factors affecting accuracy of SUV measurements. AJR Am. J. Roentgenol. 195, 310–320. 10.2214/AJR.10.4923 20651185

[B3] Aletras A. H. Tilak G. S. Natanzon A. Hsu L. Y. Gonzalez F. M. Hoyt R. F. Jr.. (2006). Retrospective determination of the area at risk for reperfused acute myocardial infarction with T2-weighted cardiac magnetic resonance imaging: histopathological and displacement encoding with stimulated echoes (DENSE) functional validations. Circulation 113, 1865–1870. 10.1161/CIRCULATIONAHA.105.576025 16606793

[B4] Alsaid H. Bao W. Rambo M. V. Logan G. A. Figueroa D. J. Lenhard S. C.. (2012). Serial MRI characterization of the functional and morphological changes in mouse lung in response to cardiac remodeling following myocardial infarction. Magn. Reson. Med. 67, 191–200. 10.1002/mrm.22973 21671268

[B5] Alzoubi A. Toba M. Abe K. O'Neill K. D. Rocic P. Fagan K. A.. (2013). Dehydroepiandrosterone restores right ventricular structure and function in rats with severe pulmonary arterial hypertension. Am. J. Physiol. Heart Circ. Physiol. 304, H1708–H1718. 10.1152/ajpheart.00746.2012 23585128

[B6] Awan Z. Denis M. Bailey D. Giaid A. Prat A. Goltzman D.. (2011). The LDLR deficient mouse as a model for aortic calcification and quantification by micro-computed tomography. Atherosclerosis 219, 455–462. 10.1016/j.atherosclerosis.2011.08.035 22051553

[B7] Babiker F. A. Lips D. J. Delvaux E. Zandberg P. Janssen B. J. Prinzen F.. (2007). Oestrogen modulates cardiac ischaemic remodelling through oestrogen receptor-specific mechanisms. Acta Physiol. (Oxf). 189, 23–31. 10.1111/j.1748-1716.2006.01633.x 17280554

[B8] Badimon L. (2001). Atherosclerosis and thrombosis: lessons from animal models. Thromb. Haemost. 86, 356–365. 11487025

[B9] Bao W. Aravindhan K. Alsaid H. Chendrimada T. Szapacs M. Citerone D. R.. (2011). Albiglutide, a long lasting glucagon-like peptide-1 analog, protects the rat heart against ischemia/reperfusion injury: evidence for improving cardiac metabolic efficiency. PLoS ONE 6:e23570. 10.1371/journal.pone.0023570 21887274 PMC3162574

[B10] Bao W. Ballard V. L. Needle S. Hoang B. Lenhard S. C. Tunstead J. R.. (2013). Cardioprotection by systemic dosing of thymosin beta four following ischemic myocardial injury. Front. Pharmacol. 4:149. 10.3389/fphar.2013.00149 24348421 PMC3843122

[B11] Basso C. Fox P. R. Meurs K. M. Towbin J. A. Spier A. W. Calabrese F.. (2004). Arrhythmogenic right ventricular cardiomyopathy causing sudden cardiac death in boxer dogs: a new animal model of human disease. Circulation 109, 1180–1185. 10.1161/01.CIR.0000118494.07530.65 14993138

[B12] Bauer M. Cheng S. Jain M. Ngoy S. Theodoropoulos C. Trujillo A.. (2011). Echocardiographic speckle-tracking based strain imaging for rapid cardiovascular phenotyping in mice. Circ. Res. 108, 908–916. 10.1161/CIRCRESAHA.110.239574 21372284 PMC3376717

[B13] Bengel F. M. Higuchi T. Javadi M. S. Lautamäki R. (2009). Cardiac positron emission tomography. J. Am. Coll. Cardiol. 54, 1–15. 10.1016/j.jacc.2009.02.065 19555834

[B14] Bers D. M. (2001). Control of cardiac contraction by SR and sarcolemmal Ca fluxes, in Excitation-Contraction Coupling and Cardiac Contractile Force, Vol. 237 (Dordrecht: Springer), 245–272.

[B15] Bhattacharya P. Ross B. D. Bünger R. (2009). Cardiovascular applications of hyperpolarized contrast media and metabolic tracers. Exp. Biol. Med. (Maywood). 234, 1395–1416. 10.3181/0904-MR-135 19934362

[B16] Bianco R. W. Gallegos R. P. Rivard A. L. Voight J. Dalmasso A. P. (2009). Animal models for cardiac research, in Handbook of Cardiac Anatomy, Physiology, and Devices, 2nd Edn, ed Iaizzo P. A. (Minneapolis, MN: Springer Science + Business Media), 393–410.

[B17] Bode G. Clausing P. Gervais F. Loegsted J. Luft J. Nogues V.. (2010). The utility of the minipig as an animal model in regulatory toxicology. J. Pharmacol. Toxicol. Methods 62, 196–220. 10.1016/j.vascn.2010.05.009 20685310

[B18] Bostick B. Yue Y. Duan D. (2011). Phenotyping cardiac gene therapy in mice. Methods Mol. Biol. 709, 91–104. 10.1007/978-1-61737-982-6_6 21194023 PMC3118042

[B19] Bottomley P. A. Wu K. C. Gerstenblith G. Schulman S. P. Steinberg A. Weiss R. G. (2009). Reduced myocardial creatine kinase flux in human myocardial infarction: an *in vivo* phosphorus magnetic resonance spectroscopy study. Circulation 119, 1918–1924. 10.1161/CIRCULATIONAHA.108.823187 19332463 PMC2743337

[B20] Broisat A. Hernot S. Toczek J. De Vos J. Riou L. M. Martin S.. (2012). Nanobodies targeting mouse/human VCAM1 for the nuclear imaging of atherosclerotic lesions. Circ. Res. 110, 927–937. 10.1161/CIRCRESAHA.112.265140 22461363 PMC3918224

[B21] Bryson-Richardson R. J. Berger S. Schilling T. F. Hall T. E. Cole N. J. Gibson A. J.. (2007). FishNet: an online database of zebrafish anatomy. BMC Biol. 5:34. 10.1186/1741-7007-5-34 17705855 PMC2031877

[B22] Bunck A. C. Engelen M. A. Schnackenburg B. Furkert J. Bremer C. Heindel W.. (2009). Feasibility of functional cardiac MR imaging in mice using a clinical 3 Tesla whole body scanner. Invest. Radiol. 44, 749–756. 10.1097/RLI.0b013e3181b2c135 19838122

[B23] Carlsson M. Ubachs J. F. Hedström E. Heiberg E. Jovinge S. Arheden H. (2009). Myocardium at risk after acute infarction in humans on cardiac magnetic resonance: quantitative assessment during follow-up and validation with single-photon emission computed tomography. JACC Cardiovasc. Imaging 2, 569–576. 10.1016/j.jcmg.2008.11.018 19442942

[B24] Cassidy P. J. Schneider J. E. Grieve S. M. Lygate C. Neubauer S. Clarke K. (2004). Assessment of motion gating strategies for mouse magnetic resonance at high magnetic fields. J. Magn. Resonance Imaging 19, 229–237. 10.1002/jmri.10454 14745758

[B25] Ciuclan L. Bonneau O. Hussey M. Duggan N. Holmes A. M. Good R.. (2011). A novel murine model of severe pulmonary arterial hypertension. Am. J. Respir. Crit. Care Med. 184, 1171–1182. 10.1164/rccm.201103-0412OC 21868504

[B26] Constantinides C. (2013). Study of the murine cardiac mechanical function using magnetic resonance imaging: the current status, challenges, and future perspectives, in Practical Applications in Biomedical Engineering, eds Andrade A. A. P. A. O. Naves E. L. M. Soares A. B. (Rijeka: InTech). 10.5772/51364

[B27] Coolen B. F. Geelen T. Paulis L. E. Nicolay K. Strijkers G. J. (2011). Regional contrast agent quantification in a mouse model of myocardial infarction using 3D cardiac T1 mapping. J. Cardiovasc. Magn. Reson. 13:56. 10.1186/1532-429X-13-56 21974927 PMC3207957

[B28] Cordeiro M. A. S. Lima J. A. C. (2006). Atherosclerotic plaque characterization by multidetector row computed tomography angiography. J. Am. Coll. Cardiol. 47(8 Suppl.), C40–C47. 10.1016/j.jacc.2005.09.076 16631509

[B29] Corti R. Fuster V. (2011). Imaging of atherosclerosis: magnetic resonance imaging. Eur. Heart J. 32, 1709b–1719b. 10.1093/eurheartj/ehr068 21508002

[B30] Crick S. J. Sheppard M. N. Ho S. Y. Gebstein L. Anderson R. H. (1998). Anatomy of the pig heart: comparisons with normal human cardiac structure. J. Anat. 193(Pt 1), 105–119. 10.1046/j.1469-7580.1998.19310105.x 9758141 PMC1467827

[B31] Cruz F. M. Sanz-Rosa D. Roche-Molina M. García-Prieto J. García-Ruiz J. M. Pizarro G.. (2015). Exercise triggers ARVC phenotype in mice expressing a disease-causing mutated version of human plakophilin-2. J. Am. Coll. Cardiol. 65, 1438–1450. 10.1016/j.jacc.2015.01.045 25857910

[B32] Chappell D. Heindl B. Jacob M. Annecke T. Chen C. Rehm M.. (2011). Sevoflurane reduces leukocyte and platelet adhesion after ischemia-reperfusion by protecting the endothelial glycocalyx. Anesthesiology 115, 483–491. 10.1097/ALN.0b013e3182289988 21785339

[B33] Checovich W. J. Fitch W. L. Krauss R. M. Smith M. P. Rapacz J. Smith C. L.. (1988). Defective catabolism and abnormal composition of low-density lipoproteins from mutant pigs with hypercholesterolemia. Biochemistry 27, 1934–1941. 10.1021/bi00406a020 3378039

[B34] Chen G. Li Y. Tian J. Zhang L. Jean-Charles P. Gobara N.. (2012). Application of echocardiography on transgenic mice with cardiomyopathies. Biochem. Res. Int. 2012, 715197–715199. 10.1155/2012/715197 22675635 PMC3363992

[B35] Cheng H.-W. Fisch S. Cheng S. Bauer M. Ngoy S. Qiu Y.. (2014). Assessment of right ventricular structure and function in mouse model of pulmonary artery constriction by transthoracic echocardiography. J. Vis. Exp. 84:e51041. 10.3791/51041 24513696 PMC4397999

[B36] Chien K. R. (1996). Genes and physiology: molecular physiology in genetically engineered animals. J. Clin. Invest. 97, 901–909. 10.1172/JCI118512 8613542 PMC507134

[B37] Chung J. Liu H. Jeong E.-M. Gu L. Gladstein S. Farzaneh-Far A. (2013). *In vivo* validation of an ultra-high field, high temporal resolution myocardial tagging technique for assessment of diastolic function in mice. J. Cardiovasc. Magn. Reson. 15(Suppl. 1), P129 10.1186/1532-429X-15-S1-P129

[B38] Davies J. R. Rudd J. H. F. Fryer T. D. Graves M. J. Clark J. C. Kirkpatrick P. J.. (2005). Identification of culprit lesions after transient ischemic attack by combined 18F fluorodeoxyglucose positron-emission tomography and high-resolution magnetic resonance imaging. Stroke 36, 2642–2647. 10.1161/01.STR.0000190896.67743.b1 16282536

[B39] de La Roque E. D. Thiaudière E. Ducret T. Marthan R. Franconi J. M. Guibert C.. (2011). Effect of chronic hypoxia on pulmonary artery blood velocity in rats as assessed by electrocardiography-triggered three-dimensional time-resolved MR angiography. NMR Biomed. 24, 225–230. 10.1002/nbm.1574 20945307

[B40] de Raaf M. A. Schalij I. Gomez-Arroyo J. Rol N. Happé C. de Man F. S.. (2014). SuHx rat model: partly reversible pulmonary hypertension and progressive intima obstruction. Eur. Respir. J. 44, 160–168. 10.1183/09031936.00204813 24791833

[B41] Di Carli M. F. Dorbala S. Meserve J. El Fakhri G. Sitek A. Moore S. C. (2007). Clinical myocardial perfusion PET/CT. J. Nucl. Med. 48, 783–793. 10.2967/jnumed.106.032789 17475968

[B42] Dilsizian V. Bacharach S. L. Beanlands R. S. Bergmann S. R. Delbeke D. Gropler R. J. (2009). PET myocardial perfusion and metabolism clinical imaging. J. Nucl. Cardiol. 16, 651–651. 10.1007/s12350-009-9094-9

[B43] Dixon J. A. Spinale F. G. (2009). Large animal models of heart failure: a critical link in the translation of basic science to clinical practice. Circ. Heart Fail. 2, 262–271. 10.1161/CIRCHEARTFAILURE.108.814459 19808348 PMC2762217

[B44] Dixon J. L. Stoops J. D. Parker J. L. Laughlin M. H. Weisman G. A. Sturek M. (1999). Dyslipidemia and vascular dysfunction in diabetic pigs fed an atherogenic diet. Arterioscler. Thromb. Vasc. Biol. 19, 2981–2992. 10.1161/01.ATV.19.12.2981 10591679

[B45] Doevendans P. A. Daemen M. J. de Muinck E. D. Smits J. F. (1998). Cardiovascular phenotyping in mice. Cardiovasc. Res. 39, 34–49. 10.1016/S0008-6363(98)00073-X 9764188

[B46] Doevendans P. A. Hunter J. J. Lembo G. Wollert K. C. (1995). Strategies for studying cardiovascular diseases in transgenic mice and gene-targeted mice, in Transgenic Animal Science, ed Monastersky R. J. (Washington, DC: American Socity of Microbiology), 107–144.

[B47] Dumas de La Roque E. Bellance N. Rossignol R. Begueret H. Billaud M. dos Santos P.. (2012). Dehydroepiandrosterone reverses chronic hypoxia/reoxygenation-induced right ventricular dysfunction in rats. Eur. Respir. J. 40, 1420–1429. 10.1183/09031936.00011511 22523357

[B48] Epstein F. H. Yang Z. Gilson W. D. Berr S. S. Kramer C. M. French B. A. (2002). MR tagging early after myocardial infarction in mice demonstrates contractile dysfunction in adjacent and remote regions. Magn. Reson. Med. 48, 399–403. 10.1002/mrm.10210 12210951

[B49] Espe E. K. Aronsen J. M. Eriksen G. S. Zhang L. Smiseth O. A. Edvardsen T.. (2015). Assessment of regional myocardial work in rats. Circ. Cardiovasc. Imaging 8:e002695. 10.1161/CIRCIMAGING.114.002695 25673647

[B50] Fayad Z. A. Fallon J. T. Shinnar M. Wehrli S. Dansky H. M. Poon M.. (1998). Noninvasive *in vivo* high-resolution magnetic resonance imaging of atherosclerotic lesions in genetically engineered mice. Circulation 98, 1541–1547. 10.1161/01.CIR.98.15.1541 9769308

[B51] Fayssoil A. Tournoux F. (2013). Analyzing left ventricular function in mice with Doppler echocardiography. Heart Fail. Rev. 18, 511–516. 10.1007/s10741-012-9345-8 22961495

[B52] Feiring A. J. Johnson M. R. Kioschos J. M. Kirchner P. T. Marcus M. L. White C. W. (1987). The importance of the determination of the myocardial area at risk in the evaluation of the outcome of acute myocardial infarction in patients. Circulation 75, 980–987. 10.1161/01.CIR.75.5.980 3568313

[B53] Ferferieva V. Van den Bergh A. Claus P. Jasaityte R. La Gerche A. Rademakers F.. (2013). Assessment of strain and strain rate by two-dimensional speckle tracking in mice: comparison with tissue Doppler echocardiography and conductance catheter measurements. Eur. Heart J. Cardiovasc. Imaging 14, 765–773. 10.1093/ehjci/jes274 23209279

[B54] Fernández-Friera L. García-Álvarez A. Ibáñez B. (2013). Imaginando el futuro del diagnóstico por imagen. Rev. Esp. Cardiol. 66, 134–143. 10.1016/j.recesp.2012.10.012 24775390

[B55] Fernández-Jiménez R. García-Prieto J. Sánchez-González J. Agüero J. López-Martín G. J. Galán-Arriola C.. (2015a). Pathophysiology Underlying the Bimodal Edema Phenomenon After Myocardial Ischemia/Reperfusion. J. Am. Coll. Cardiol. 66, 816–828. 10.1016/j.jacc.2015.06.023 26271065

[B56] Fernandez-Jimenez R. Sanchez-Gonzalez J. Aguero J. Garcia-Prieto J. Lopez-Martin G. J. Garcia-Ruiz J. M. (2015b). Myocardial edema after ischemia/reperfusion is not stable and follows a bimodal pattern: imaging and histological tissue characterization. J. Am. Coll. Cardiol. 65, 315–323. 10.1016/j.jacc.2014.11.004 25460833

[B57] Finsen A. V. Christensen G. Sjaastad I. (2005). Echocardiographic parameters discriminating myocardial infarction with pulmonary congestion from myocardial infarction without congestion in the mouse. J. Appl. Physiol. 98, 680–689. 10.1152/japplphysiol.00924.2004 15475595

[B58] Foryst-Ludwig A. Kreissl M. C. Sprang C. Thalke B. Böhm C. Benz V.. (2011). Sex differences in physiological cardiac hypertrophy are associated with exercise-mediated changes in energy substrate availability. Am. J. Physiol. Heart Circ. Physiol. 301, H115–H122. 10.1152/ajpheart.01222.2010 21478409

[B59] Franco F. Thomas G. D. Giroir B. Bryant D. Bullock M. C. Chwialkowski M. C.. (1999). Magnetic resonance imaging and invasive evaluation of development of heart failure in transgenic mice with myocardial expression of tumor necrosis factor-alpha. Circulation 99, 448–454. 10.1161/01.CIR.99.3.448 9918534

[B60] Friedrich M. G. Abdel-Aty H. Taylor A. Schulz-Menger J. Messroghli D. Dietz R. (2008). The salvaged area at risk in reperfused acute myocardial infarction as visualized by cardiovascular magnetic resonance. J. Am. Coll. Cardiol. 51, 1581–1587. 10.1016/j.jacc.2008.01.019 18420102

[B61] Fuster J. J. Castillo A. I. Zaragoza C. Ibáñez B. Andrés V. (2012). Animal models of atherosclerosis. Prog. Mol. Biol. Transl. Sci. 105, 1–23. 10.1016/B978-0-12-394596-9.00001-9 22137427

[B62] Gao X. M. Dart A. M. Dewar E. Jennings G. Du X. J. (2000). Serial echocardiographic assessment of left ventricular dimensions and function after myocardial infarction in mice. Cardiovasc. Res. 45, 330–338. 10.1016/S0008-6363(99)00274-6 10728353

[B63] García-Álvarez A. Fernández-Friera L. García-Ruiz J. M. Nuño-Ayala M. Pereda D. Fernández-Jiménez R.. (2013). Noninvasive monitoring of serial changes in pulmonary vascular resistance and acute vasodilator testing using cardiac magnetic resonance. J. Am. Coll. Cardiol. 62, 1621–1631. 10.1016/j.jacc.2013.07.037 23954344

[B64] García-Álvarez A. García-Lunar I. Pereda D. Fernández-Jimenez R. Sánchez-González J. Mirelis J. G.. (2015). Association of myocardial T1-mapping CMR with hemodynamics and RV performance in pulmonary hypertension. JACC Cardiovasc. Imaging 8, 76–82. 10.1016/j.jcmg.2014.08.012 25592698

[B65] Gargiulo S. Greco A. Gramanzini M. Esposito S. Affuso A. Brunetti A.. (2012a). Mice anesthesia, analgesia, and care, Part II: anesthetic considerations in preclinical imaging studies. ILAR J. 53, E70–E81. 10.1093/ilar.53.1.70 23382272

[B66] Gargiulo S. Greco A. Gramanzini M. Petretta M. P. Ferro A. Larobina M.. (2012b). PET/CT imaging in mouse models of myocardial ischemia. J. Biomed. Biotechnol. 2012, 541872–541812. 10.1155/2012/541872 22505813 PMC3312322

[B67] Ge Z. D. Pravdic D. Bienengraeber M. Pratt P. F. Jr. Auchampach J. A. Gross G. J.. (2010). Isoflurane postconditioning protects against reperfusion injury by preventing mitochondrial permeability transition by an endothelial nitric oxide synthase-dependent mechanism. Anesthesiology 112, 73–85. 10.1097/ALN.0b013e3181c4a607 19996950 PMC4374483

[B68] Gerrity R. G. Natarajan R. Nadler J. L. Kimsey T. (2001). Diabetes-induced accelerated atherosclerosis in swine. Diabetes 50, 1654–1665. 10.2337/diabetes.50.7.1654 11423488

[B69] Giannarelli C. Ibanez B. Cimmino G. Garcia Ruiz J. M. Faita F. Bianchini E.. (2010). Contrast-enhanced ultrasound imaging detects intraplaque neovascularization in an experimental model of atherosclerosis. JACC Cardiovasc. Imaging 3, 1256–1264. 10.1016/j.jcmg.2010.09.017 21163454

[B70] Gilson W. D. Kraitchman D. L. (2007). Cardiac magnetic resonance imaging in small rodents using clinical 1.5 T and 3.0 T scanners. Methods 43, 35–45. 10.1016/j.ymeth.2007.03.012 17720562 PMC2075472

[B71] Gomez-Arroyo J. G. Farkas L. Alhussaini A. A. Farkas D. Kraskauskas D. Voelkel N. F.. (2012). The monocrotaline model of pulmonary hypertension in perspective. Am. J. Physiol. Lung Cell. Mol. Physiol. 302, L363–L369. 10.1152/ajplung.00212.2011 21964406

[B72] González-Rosa J. M. Guzmán-Martínez G. Marques I. J. Sánchez-Iranzo H. Jiménez-Borreguero L. J. Mercader N. (2014). Use of echocardiography reveals reestablishment of ventricular pumping efficiency and partial ventricular wall motion recovery upon ventricular cryoinjury in the zebrafish. PLoS ONE 9:e115604. 10.1371/journal.pone.0115604 25532015 PMC4274112

[B73] Grieve S. M. Lønborg J. Mazhar J. Tan T. C. Ho E. Liu C. C.. (2013). Cardiac magnetic resonance imaging of rapid VCAM-1 up-regulation in myocardial ischemia-reperfusion injury. Eur. Biophys. J. 42, 61–70. 10.1007/s00249-012-0857-x 23052973

[B74] Groten T. Pierce A. A. Huen A. C. Schnaper H. W. (2005). 17 β-estradiol transiently disrupts adherens junctions in endothelial cells. FASEB J 19, 1368–1370. 10.1096/fj.04-2558fje 15928195

[B75] Hasenfuss G. (1998). Animal models of human cardiovascular disease, heart failure and hypertrophy. Cardiovasc. Res. 39, 60–76. 10.1016/S0008-6363(98)00110-2 9764190

[B76] Hearse D. J. Sutherland F. J. (2000). Experimental models for the study of cardiovascular function and disease. Pharmacol. Res. 41, 597–603. 10.1006/phrs.1999.0651 10816328

[B77] Helft G. Worthley S. G. Fuster V. Fayad Z. A. Zaman A. G. Corti R.. (2002). Progression and regression of atherosclerotic lesions: monitoring with serial noninvasive magnetic resonance imaging. Circulation 105, 993–998. 10.1161/hc0802.104325 11864931

[B78] Helft G. Worthley S. G. Fuster V. Zaman A. G. Schechter C. Osende J. I.. (2001). Atherosclerotic aortic component quantification by noninvasive magnetic resonance imaging: an *in vivo* study in rabbits. J. Am. Coll. Cardiol. 37, 1149–1154. 10.1016/S0735-1097(01)01141-X 11263622

[B79] Hill J. A. Iaizzo P. A. (2009). Comparative cardiac anatomy, in Handbook of Cardiac Anatomy, Physiology, and Devices, ed Iaizzo P. A. (Totowa, NJ: Springer Science & Business Media), 87–108. 10.1007/978-1-60327-372-5_6

[B80] Ho C. Y. Solomon S. D. (2006). A clinician's guide to tissue Doppler imaging. Circulation 113, e396–e398. 10.1161/CIRCULATIONAHA.105.579268 16534017

[B81] Houser S. R. Margulies K. B. Murphy A. M. Spinale F. G. Francis G. S. Prabhu S. D.. (2012). Animal models of heart failure: a scientific statement from the American Heart Association. Circ. Res. 111, 131–150. 10.1161/RES.0b013e3182582523 22595296

[B82] Hoyt R. E. Hawkins J. V. St Clair M. B. Kennett M. J. (2006). Mouse physiology, in The Mouse in Biomedical Research, eds Fox J. G. Davisson M. T. Quimby F. W. Barthold S. W. Newcomer C. E. Smith A. L. (Burlington, MA: Academic Press III), 23–90.

[B83] Ibanez B. Giannarelli C. Cimmino G. Santos-Gallego C. G. Alique M. Pinero A.. (2012). Recombinant HDL(Milano) exerts greater anti-inflammatory and plaque stabilizing properties than HDL(wild-type). Atherosclerosis 220, 72–77. 10.1016/j.atherosclerosis.2011.10.006 22030095

[B84] Ibanez B. Prat-González S. Speidl W. S. Vilahur G. Pinero A. Cimmino G.. (2007). Early metoprolol administration before coronary reperfusion results in increased myocardial salvage: analysis of ischemic myocardium at risk using cardiac magnetic resonance. Circulation 115, 2909–2916. 10.1161/CIRCULATIONAHA.106.679639 17515460

[B85] Ignatowski A. (1908). Changes in parenchymatous organs and in the aorta of rabbits under the influence of animal protein. Izvestia Imperatorskoi Voenno-Medicinskoi Akademii 18, 231–244.

[B86] James J. F. Hewett T. E. Robbins J. (1998). Cardiac physiology in transgenic mice. Circ. Res. 82, 407–415. 10.1161/01.RES.82.4.407 9506700

[B87] Johnson M. S. Moore R. L. Brown D. A. (2006). Sex differences in myocardial infarct size are abolished by sarcolemmal KATP channel blockade in rat. Am. J. Physiol. Heart Circ. Physiol. 290, H2644–H2647. 10.1152/ajpheart.01291.2005 16473955

[B88] Kass D. A. Hare J. M. Georgakopoulos D. (1998). Murine cardiac function: a cautionary tail. Circ. Res. 82, 519–522. 10.1161/01.RES.82.4.519 9506713

[B89] Kellman P. Aletras A. H. Mancini C. McVeigh E. R. Arai A. E. (2007). T2-prepared SSFP improves diagnostic confidence in edema imaging in acute myocardial infarction compared to turbo spin echo. Magn. Reson. Med. 57, 891–897. 10.1002/mrm.21215 17457880 PMC2396276

[B90] Kelly K. A. Allport J. R. Tsourkas A. Shinde-Patil V. R. Josephson L. Weissleder R. (2005). Detection of vascular adhesion molecule-1 expression using a novel multimodal nanoparticle. Circ. Res. 96, 327–336. 10.1161/01.RES.0000155722.17881.dd 15653572

[B91] Kim R. J. Fieno D. S. Parrish T. B. Harris K. Chen E. L. Simonetti O.. (1999). Relationship of MRI delayed contrast enhancement to irreversible injury, infarct age, and contractile function. Circulation 100, 1992–2002. 10.1161/01.CIR.100.19.1992 10556226

[B92] Kittleson M. D. Meurs K. M. Munro M. J. Kittleson J. A. Liu S. K. Pion P. D.. (1999). Familial hypertrophic cardiomyopathy in maine coon cats: an animal model of human disease. Circulation 99, 3172–3180. 10.1161/01.CIR.99.24.3172 10377082

[B93] Knuuti M. J. Nuutila P. Ruotsalainen U. Saraste M. Harkonen R. Ahonen A.. (1992). Euglycemic hyperinsulinemic clamp and oral glucose load in stimulating myocardial glucose utilization during positron emission tomography. J. Nucl. Med. 33, 1255–1262. 1613561

[B94] Kober F. Iltis I. Cozzone P. J. Bernard M. (2005). Myocardial blood flow mapping in mice using high-resolution spin labeling magnetic resonance imaging: influence of ketamine/xylazine and isoflurane anesthesia. Magn. Reson. Med. 53, 601–606. 10.1002/mrm.20373 15723407

[B95] Koktzoglou I. Harris K. R. Tang R. Kane B. J. Misselwitz B. Weinmann H.-J.. (2006). Gadofluorine-enhanced magnetic resonance imaging of carotid atherosclerosis in Yucatan miniswine. Invest. Radiol. 41, 299–304. 10.1097/01.rli.0000188362.12555.62 16481913

[B96] Laber K. E. Whary M. T. Bingel S. A. (2002). Biology and diseases of swine, Biology and Diseases of Swine, eds Fox J. G. Anderson L. C. Loew F. M. Quimby F. W. (Waltham, MA: Laboratory Animal), 616–665. 10.1016/b978-012263951-7/50018-1

[B97] Laflamme M. A. Murry C. E. (2011). Heart regeneration. Nature 473, 326–335. 10.1038/nature10147 21593865 PMC4091722

[B98] Lautamäki R. Schuleri K. H. Sasano T. Javadi M. S. Youssef A. Merrill J.. (2009). Integration of infarct size, tissue perfusion, and metabolism by hybrid cardiac positron emission tomography/computed tomography: evaluation in a porcine model of myocardial infarction. Circ. Cardiovasc. Imaging 2, 299–305. 10.1161/CIRCIMAGING.108.846253 19808610

[B99] Lee Y. A. Kim J. I. Lee J. W. Cho Y. J. Lee B. H. Chung H. W.. (2012). Effects of various anesthetic protocols on 18F-flurodeoxyglucose uptake into the brains and hearts of normal miniature pigs (Sus scrofa domestica). J. Am. Assoc. Lab. Anim. Sci. 51, 246–252. 22776126 PMC3314529

[B100] Lelovas P. P. Kostomitsopoulos N. G. Xanthos T. T. (2014). A comparative anatomic and physiologic overview of the porcine heart. J. Am. Assoc. Lab. Anim. Sci. 53, 432–438. 25255064 PMC4181683

[B101] Levin C. S. Zaidi H. (2007). Current Trends in Preclinical PET System Design. PET Clin. 2, 125–160. 10.1016/j.cpet.2007.12.001 27157870

[B102] Libby P. (2002). Inflammation in atherosclerosis. Nature 420, 868–874. 10.1038/nature01323 12490960

[B103] Linardi D. Rungatscher A. Morjan M. Marino P. Luciani G. B. Mazzucco A.. (2014). Ventricular and pulmonary vascular remodeling induced by pulmonary overflow in a chronic model of pretricuspid shunt. J. Thorac. Cardiovasc. Surg. 148, 2609–2617. 10.1016/j.jtcvs.2014.04.044 24908349

[B104] Loganathan R. Bilgen M. Al-Hafez B. Alenezy M. D. Smirnova I. V. (2006). Cardiac dysfunction in the diabetic rat: quantitative evaluation using high resolution magnetic resonance imaging. Cardiovasc. Diabetol. 5:7. 10.1186/1475-2840-5-7 16595006 PMC1450259

[B105] Lompre A. M. Mercadier J. J. Wisnewsky C. Bouveret P. Pantaloni C. D'Albis A.. (1981). Species- and age-dependent changes in the relative amounts of cardiac myosin isoenzymes in mammals. Dev. Biol. 84, 286–290. 10.1016/0012-1606(81)90396-1 20737866

[B106] Lutgens E. Daemen M. J. de Muinck E. D. Debets J. Leenders P. Smits J. F. (1999). Chronic myocardial infarction in the mouse: cardiac structural and functional changes. Cardiovasc. Res. 41, 586–593. 10.1016/S0008-6363(98)00216-8 10435030

[B107] Lloyd-Jones D. Adams R. J. Brown T. M. Carnethon M. Dai S. De Simone G.. (2010). Executive summary: heart disease and stroke statistics–2010 update: a report from the American Heart Association. Circulation 121, 948–954. 10.1161/CIRCULATIONAHA.109.192666 20177011

[B108] Mahmoodzadeh S. Dworatzek E. Fritschka S. Pham T. H. Regitz-Zagrosek V. (2010). 17beta-Estradiol inhibits matrix metalloproteinase-2 transcription via MAP kinase in fibroblasts. Cardiovasc. Res. 85, 719–728. 10.1093/cvr/cvp350 19861308 PMC2819834

[B109] Mahmoodzadeh S. Fliegner D. Dworatzek E. (2012). Sex differences in animal models for cardiovascular diseases and the role of estrogen. Handb. Exp. Pharmacol. 214, 23–48. 10.1007/978-3-642-30726-3_2 23027444

[B110] Makowski M. R. Wiethoff A. J. Jansen C. H. Botnar R. M. (2010). Cardiovascular MRI in small animals. Expert Rev. Cardiovasc. Ther. 8, 35–47. 10.1586/erc.09.126 20014933

[B111] Mann D. L. Bristow M. R. (2005). Mechanisms and models in heart failure: the biomechanical model and beyond. Circulation 111, 2837–2849. 10.1161/CIRCULATIONAHA.104.500546 15927992

[B112] Matthews P. M. Coatney R. Alsaid H. Jucker B. Ashworth S. Parker C.. (2013). Technologies: preclinical imaging for drug development. Drug Discov. Today Technol. 10, e343–e350. 10.1016/j.ddtec.2012.04.004 24050130

[B113] McKellar S. H. Javan H. Bowen M. E. Liu X. Schaaf C. L. Briggs C. M.. (2015). Animal model of reversible, right ventricular failure. J. Surg. Res. 194, 327–333. 10.1016/j.jss.2014.11.006 25541238

[B114] McLean M. Prothero J. (1992). Determination of relative fiber orientation in heart muscle: methodological problems. Anat. Rec. 232, 459–465. 10.1002/ar.1092320402 1554098

[B115] Meerson F. Z. (1961). On the mechanism of compensatory hyperfunction and insufficiency of the heart. Cor Vasa 3, 161–177. 14472099

[B116] Meijler F. L. (1985). Atrioventricular conduction versus heart size from mouse to whale. J. Am. Coll. Cardiol. 5(2 Pt 1), 363–365. 10.1016/s0735-1097(85)80060-7 3881500

[B117] Mewton N. Rapacchi S. Augeul L. Ferrera R. Loufouat J. Boussel L.. (2011). Determination of the myocardial area at risk with pre- versus post-reperfusion imaging techniques in the pig model. Basic Res. Cardiol. 106, 1247–1257. 10.1007/s00395-011-0214-8 21874556

[B118] Michael L. H. Entman M. L. Hartley C. J. Youker K. A. Zhu J. Hall S. R.. (1995). Myocardial ischemia and reperfusion: a murine model. Am. J. Physiol. 269(6 Pt 2), H2147–H2154. 8594926 10.1152/ajpheart.1995.269.6.H2147

[B119] Michalska M. Machtoub L. Manthey H. D. Bauer E. Herold V. Krohne G.. (2012). Visualization of vascular inflammation in the atherosclerotic mouse by ultrasmall superparamagnetic iron oxide vascular cell adhesion molecule-1-specific nanoparticles. Arterioscler. Thromb. Vasc. Biol. 32, 2350–2357. 10.1161/ATVBAHA.112.255224 22879583

[B120] Millon A. Dickson S. D. Klink A. Izquierdo-Garcia D. Bini J. Lancelot E.. (2013). Monitoring plaque inflammation in atherosclerotic rabbits with an iron oxide (P904) and (18)F-FDG using a combined PET/MR scanner. Atherosclerosis 228, 339–345. 10.1016/j.atherosclerosis.2013.03.019 23582588 PMC4128694

[B121] Mor-Avi V. Lang R. M. Badano L. P. Belohlavek M. Cardim N. M. Derumeaux G.. (2011). Current and evolving echocardiographic techniques for the quantitative evaluation of cardiac mechanics: ASE/EAE consensus statement on methodology and indications endorsed by the Japanese Society of Echocardiography. Eur. J. Echocardiogr. 12, 167–205. 10.1093/ejechocard/jer021 21385887

[B122] Moran C. M. Thomson A. J. W. Rog-Zielinska E. Gray G. A. (2013). High-resolution echocardiography in the assessment of cardiac physiology and disease in preclinical models. Exp. Physiol. 98, 629–644. 10.1113/expphysiol.2012.068577 23118017

[B123] Mulder W. J. Jaffer F. A. Fayad Z. A. Nahrendorf M. (2014). Imaging and nanomedicine in inflammatory atherosclerosis. Sci. Transl. Med. 6, 239sr231. 10.1126/scitranslmed.3005101 24898749 PMC4110972

[B124] Nahrendorf M. Jaffer F. A. Kelly K. A. Sosnovik D. E. Aikawa E. Libby P.. (2006). Noninvasive vascular cell adhesion molecule-1 imaging identifies inflammatory activation of cells in atherosclerosis. Circulation 114, 1504–1511. 10.1161/CIRCULATIONAHA.106.646380 17000904

[B125] Nahrendorf M. Keliher E. Panizzi P. Zhang H. Hembrador S. Figueiredo J. L.. (2009). 18F-4V for PET-CT imaging of VCAM-1 expression in atherosclerosis. JACC Cardiovasc. Imaging 2, 1213–1222. 10.1016/j.jcmg.2009.04.016 19833312 PMC2773129

[B126] Nakashima Y. Plump A. S. Raines E. W. Breslow J. L. Ross R. (1994). ApoE-deficient mice develop lesions of all phases of atherosclerosis throughout the arterial tree. Arterioscler. Thromb. 14, 133–140. 10.1161/01.ATV.14.1.133 8274468

[B127] Nakata T. M. Tanaka R. Yoshiyuki R. Fukayama T. Goya S. Fukushima R. (2015). Effects of single drug and combined short-term administration of sildenafil, pimobendan, and nicorandil on right ventricular function in rats with monocrotaline-induced pulmonary hypertension. J. Cardiovasc. Pharmacol. 65, 640–648. 10.1097/FJC.0000000000000236 25806612 PMC4461396

[B128] Nekolla S. G. Martinez-Moeller A. Saraste A. (2009). PET and MRI in cardiac imaging: from validation studies to integrated applications. Eur. J. Nucl. Med. Mol. Imaging 36 (Suppl. 1), S121–S130. 10.1007/s00259-008-0980-1 19104798

[B129] Nichols M. Townsend N. Scarborough P. Rayner M. (2014). Cardiovascular disease in Europe 2014: epidemiological update. Eur. Heart J. 35, 2950–2959. 10.1093/eurheartj/ehu299 25139896

[B130] Nitta-Seko A. Nitta N. Shiomi M. Sonoda A. Ota S. Tsuchiya K.. (2010). Utility of contrast-enhanced ultrasonography for qualitative imaging of atherosclerosis in Watanabe heritable hyperlipidemic rabbits: initial experimental study. Jpn. J. Radiol. 28, 656–662. 10.1007/s11604-010-0487-0 21113749

[B131] Okumura K. Kato H. Honjo O. Breitling S. Kuebler W. M. Sun M.. (2015). Carvedilol improves biventricular fibrosis and function in experimental pulmonary hypertension. J. Mol. Med. 93, 663–674. 10.1007/s00109-015-1251-9 25595602

[B132] Owen D. R. Lindsay A. C. Choudhury R. P. Fayad Z. A. (2011). Imaging of atherosclerosis. Annu. Rev. Med. 62, 25–40. 10.1146/annurev-med-041709-133809 21226610 PMC4041162

[B133] Packard R. R. S. Huang S.-C. Dahlbom M. Czernin J. Maddahi J. (2014). Absolute quantitation of myocardial blood flow in human subjects with or without myocardial ischemia using dynamic flurpiridaz F 18 PET. J. Nucl. Med. 55, 1438–1444. 10.2967/jnumed.114.141093 25071096 PMC4315668

[B134] Palermo V. Stafford Johnson M. J. Sala E. Brambilla P. G. Martin M. W. (2011). Cardiomyopathy in Boxer dogs: a retrospective study of the clinical presentation, diagnostic findings and survival. J. Vet. Cardiol. 13, 45–55. 10.1016/j.jvc.2010.06.005 21306968

[B135] Patten R. D. Hall-Porter M. R. (2009). Small animal models of heart failure: development of novel therapies, past and present. Circ. Heart Fail. 2, 138–144. 10.1161/CIRCHEARTFAILURE.108.839761 19808329

[B136] Patten R. D. Pourati I. Aronovitz M. J. Alsheikh-Ali A. Eder S. Force T.. (2008). 17 Beta-estradiol differentially affects left ventricular and cardiomyocyte hypertrophy following myocardial infarction and pressure overload. J. Card. Fail. 14, 245–253. 10.1016/j.cardfail.2007.10.024 18381189 PMC4181711

[B137] Patten R. D. Pourati I. Aronovitz M. J. Baur J. Celestin F. Chen X.. (2004). 17beta-estradiol reduces cardiomyocyte apoptosis *in vivo* and *in vitro* via activation of phospho-inositide-3 kinase/Akt signaling. Circ. Res. 95, 692–699. 10.1161/01.RES.0000144126.57786.89 15345655

[B138] Pereda D. García-Alvarez A. Sánchez-Quintana D. Nuño M. Fernández-Friera L. Fernández-Jiménez R.. (2014). Swine model of chronic postcapillary pulmonary hypertension with right ventricular remodeling: long-term characterization by cardiac catheterization, magnetic resonance, and pathology. J. Cardiovasc. Transl. Res. 7, 494–506. 10.1007/s12265-014-9564-6 24771313

[B139] Pfeffer M. A. Pfeffer J. M. Fishbein M. C. Fletcher P. J. Spadaro J. Kloner R. A.. (1979). Myocardial infarct size and ventricular function in rats. Circ. Res. 44, 503–512. 10.1161/01.RES.44.4.503 428047

[B140] Phinikaridou A. Ruberg F. L. Hallock K. J. Qiao Y. Hua N. Viereck J.. (2010). *In vivo* detection of vulnerable atherosclerotic plaque by MRI in a rabbit model. Circ. Cardiovasc. Imaging 3, 323–332. 10.1161/CIRCIMAGING.109.918524 20194634

[B141] Piao L. Fang Y. H. Cadete V. J. Wietholt C. Urboniene D. Toth P. T.. (2010). The inhibition of pyruvate dehydrogenase kinase improves impaired cardiac function and electrical remodeling in two models of right ventricular hypertrophy: resuscitating the hibernating right ventricle. J. Mol. Med. (Berl). 88, 47–60. 10.1007/s00109-009-0524-6 19949938 PMC3155251

[B142] Pohlmann A. Boye P. Wagenhaus B. Muller D. Kolanczyk M. Kohle S. (2011). Cardiac MR Imaging in Mice: Morphometry and Functional Assessment. Billerica, MA: Bruker.

[B143] Poss K. D. Wilson L. G. Keating M. T. (2002). Heart regeneration in zebrafish. Science 298, 2188–2190. 10.1126/science.1077857 12481136

[B144] Power J. M. Tonkin A. M. (1999). Large animal models of heart failure. Aust. N.Z. J. Med. 29, 395–402. 10.1111/j.1445-5994.1999.tb00734.x 10868511

[B145] Prescott M. F. McBride C. H. Hasler-Rapacz J. Von Linden J. Rapacz J. (1991). Development of complex atherosclerotic lesions in pigs with inherited hyper-LDL cholesterolemia bearing mutant alleles for apolipoprotein B. Am. J. Pathol. 139, 139–147. 1853929 PMC1886122

[B146] Ram R. Mickelsen D. M. Theodoropoulos C. Blaxall B. C. (2011). New approaches in small animal echocardiography: imaging the sounds of silence. Am. J. Physiol. Heart Circ. Physiol. 301, H1765–H1780. 10.1152/ajpheart.00559.2011 21873501 PMC3213976

[B147] Rao Y. Wang Y. L. Zhang W. S. Liu J. (2008). Emulsified isoflurane produces cardiac protection after ischemia-reperfusion injury in rabbits. Anesth. Analg. 106, 1353–1359. 10.1213/ane.0b013e3181679347 18420844

[B148] Richardson J. D. Bertaso A. G. Frost L. Psaltis P. J. Carbone A. Koschade B.. (2013). Cardiac magnetic resonance, transthoracic and transoesophageal echocardiography: a comparison of *in vivo* assessment of ventricular function in rats. Lab. Anim. 47, 291–300. 10.1177/0023677213494373 23836849

[B149] Rider O. J. Tyler D. J. (2013). Clinical implications of cardiac hyperpolarized magnetic resonance imaging. J. Cardiovasc. Magn. Reson. 15:93. 10.1186/1532-429X-15-93 24103786 PMC3819516

[B150] Roldán-Alzate A. Frydrychowicz A. Johnson K. M. Kellihan H. Chesler N. C. Wieben O.. (2014). Non-invasive assessment of cardiac function and pulmonary vascular resistance in an canine model of acute thromboembolic pulmonary hypertension using 4D flow cardiovascular magnetic resonance. J. Cardiovasc. Magn. Reson. 16:23. 10.1186/1532-429X-16-23 24625242 PMC3995608

[B151] Rottman J. N. Ni G. Khoo M. Wang Z. Zhang W. Anderson M. E.. (2003). Temporal changes in ventricular function assessed echocardiographically in conscious and anesthetized mice. J. Am. Soc. Echocardiogr. 16, 1150–1157. 10.1067/S0894-7317(03)00471-1 14608286

[B152] Ruff J. Wiesmann F. Hiller K. H. Voll S. von Kienlin M. Bauer W. R.. (1998). Magnetic resonance microimaging for noninvasive quantification of myocardial function and mass in the mouse. Magn. Reson. Med. 40, 43–48. 10.1002/mrm.1910400106 9660551

[B153] Russell J. C. Proctor S. D. (2006). Small animal models of cardiovascular disease: tools for the study of the roles of metabolic syndrome, dyslipidemia, and atherosclerosis. Cardiovasc. Pathol. 15, 318–330. 10.1016/j.carpath.2006.09.001 17113010

[B154] Ryan J. J. Archer S. L. (2014). The right ventricle in pulmonary arterial hypertension: disorders of metabolism, angiogenesis and adrenergic signaling in right ventricular failure. Circ. Res. 115, 176–188. 10.1161/CIRCRESAHA.113.301129 24951766 PMC4112290

[B155] Sánchez-González J. Fernandez-Jiménez R. Nothnagel N. D. López-Martín G. Fuster V. Ibañez B. (2015). Optimization of dual-saturation single bolus acquisition for quantitative cardiac perfusion and myocardial blood flow maps. J. Cardiovasc. Magn. Reson. 17:21. 10.1186/s12968-015-0116-2 25880970 PMC4332925

[B156] Sanz J. Fayad Z. A. (2008). Imaging of atherosclerotic cardiovascular disease. Nature 451, 953–957. 10.1038/nature06803 18288186

[B157] Schaefer A. Klein G. Brand B. Lippolt P. Drexler H. Meyer G. P. (2003). Evaluation of left ventricular diastolic function by pulsed Doppler tissue imaging in mice. J. Am. Soc. Echocardiogr. 16, 1144–1149. 10.1067/S0894-7317(03)00679-5 14608285

[B158] Schaefer A. Meyer G. P. Hilfiker-Kleiner D. Brand B. Drexler H. Klein G. (2005). Evaluation of Tissue Doppler Tei index for global left ventricular function in mice after myocardial infarction: comparison with Pulsed Doppler Tei index. Eur. J. Echocardiogr. 6, 367–375. 10.1016/j.euje.2005.01.007 16153558

[B159] Scherrer-Crosbie M. Thibault H. B. (2008). Echocardiography in translational research: of mice and men. J. Am. Soc. Echocardiogr. 21, 1083–1092. 10.1016/j.echo.2008.07.001 18723318 PMC2648388

[B160] Schmidt A. G. Gerst M. Zhai J. Carr A. N. Pater L. Kranias E. G.. (2002). Evaluation of left ventricular diastolic function from spectral and color M-mode Doppler in genetically altered mice. J. Am. Soc. Echocardiogr. 15, 1065–1073. 10.1067/mje.2002.121863 12373248

[B161] Schroeder M. A. Cochlin L. E. Heather L. C. Clarke K. Radda G. K. Tyler D. J. (2008). *In vivo* assessment of pyruvate dehydrogenase flux in the heart using hyperpolarized carbon-13 magnetic resonance. Proc. Natl. Acad. Sci. U.S.A. 105, 12051–12056. 10.1073/pnas.0805953105 18689683 PMC2515222

[B162] Simonneau G. Gatzoulis M. A. Adatia I. Celermajer D. Denton C. Ghofrani A.. (2013). Updated clinical classification of pulmonary hypertension. J. Am. Coll. Cardiol. 62(25 Suppl.), D34–D41. 10.1016/j.jacc.2013.10.029 24355639

[B163] Sinitsyn V. (2001). Magnetic resonance imaging in coronary heart disease. Eur. J. Radiol. 38, 191–199. 10.1016/S0720-048X(01)00307-2 11399372

[B164] Slawson S. E. Roman B. B. Williams D. S. Koretsky A. P. (1998). Cardiac MRI of the normal and hypertrophied mouse heart. Magn. Reson. Med. 39, 980–987. 10.1002/mrm.1910390616 9621922

[B165] Stamper D. Weissman N. J. Brezinski M. (2006). Plaque characterization with optical coherence tomography. J. Am. Coll. Cardiol. 47(8 Suppl.), C69–C79. 10.1016/j.jacc.2005.10.067 16631512

[B166] Streif J. U. Nahrendorf M. Hiller K. H. Waller C. Wiesmann F. Rommel E.. (2005). *In vivo* assessment of absolute perfusion and intracapillary blood volume in the murine myocardium by spin labeling magnetic resonance imaging. Magn. Reson. Med. 53, 584–592. 10.1002/mrm.20327 15723416

[B167] Stuckey D. J. Carr C. A. Tyler D. J. Clarke K. (2008). Cine-MRI versus two-dimensional echocardiography to measure *in vivo* left ventricular function in rat heart. NMR Biomed. 21, 765–772. 10.1002/nbm.1268 18457349

[B168] Stuckey D. J. McSweeney S. J. Thin M. Z. Habib J. Price A. N. Fiedler L. R.. (2014). T(1) mapping detects pharmacological retardation of diffuse cardiac fibrosis in mouse pressure-overload hypertrophy. Circ. Cardiovasc. Imaging 7, 240–249. 10.1161/CIRCIMAGING.113.000993 24425501

[B169] Sutendra G. Dromparis P. Paulin R. Zervopoulos S. Haromy A. Nagendran J.. (2013). A metabolic remodeling in right ventricular hypertrophy is associated with decreased angiogenesis and a transition from a compensated to a decompensated state in pulmonary hypertension. J. Mol. Med. (Berl) 91, 1315–1327. 10.1007/s00109-013-1059-4 23846254

[B170] Swindle M. M. (2007). Cardiothoracic and vascular surgery/chronic intravascular catheterization, in Swine in the Laboratory Surgery, Anesthesia, Imaging, and Experimental Techniques, ed Swindle M. M. (Boca Raton, FL: CRC Press), 195–260. 10.1201/9781420009156.ch9

[B171] Swynghedauw B. (1986). Developmental and functional adaptation of contractile proteins in cardiac and skeletal muscles. Physiol. Rev. 66, 710–771. 2942954 10.1152/physrev.1986.66.3.710

[B172] Tanaka N. Dalton N. Mao L. Rockman H. A. Peterson K. L. Gottshall K. R.. (1996). Transthoracic echocardiography in models of cardiac disease in the mouse. Circulation 94, 1109–1117. 10.1161/01.CIR.94.5.1109 8790053

[B173] Tarnavski O. McMullen J. R. Schinke M. Nie Q. Kong S. Izumo S. (2004). Mouse cardiac surgery: comprehensive techniques for the generation of mouse models of human diseases and their application for genomic studies. Physiol. Genomics 16, 349–360. 10.1152/physiolgenomics.00041.2003 14679301

[B174] Tei C. Ling L. H. Hodge D. O. Bailey K. R. Oh J. K. Rodeheffer R. J.. (1995). New index of combined systolic and diastolic myocardial performance: a simple and reproducible measure of cardiac function–a study in normals and dilated cardiomyopathy. J. Cardiol. 26, 357–366. 8558414

[B175] Temple I. P. Monfredi O. Quigley G. Schneider H. Zi M. Cartwright E. J.. (2014). Macitentan treatment retards the progression of established pulmonary arterial hypertension in an animal model. Int. J. Cardiol. 177, 423–428. 10.1016/j.ijcard.2014.09.005 25305681 PMC4251701

[B176] Teramoto N. Koshino K. Yokoyama I. Miyagawa S. Zeniya T. Hirano Y.. (2011). Experimental pig model of old myocardial infarction with long survival leading to chronic left ventricular dysfunction and remodeling as evaluated by PET. J. Nucl. Med. 52, 761–768. 10.2967/jnumed.110.084848 21498524

[B177] Thibault H. B. Kurtz B. Raher M. J. Shaik R. S. Waxman A. Derumeaux G.. (2010). Noninvasive assessment of murine pulmonary arterial pressure: validation and application to models of pulmonary hypertension. Circ. Cardiovasc. Imaging 3, 157–163. 10.1161/circimaging.109.887109 20044514 PMC3075498

[B178] Thygesen K. Alpert J. S. Jaffe A. S. Simoons M. L. Chaitman B. R. White H. D.. (2012). Third universal definition of myocardial infarction. J. Am. Coll. Cardiol. 60, 1581–1598. 10.1016/j.jacc.2012.08.001 22958960

[B179] Tian J. Hu S. Sun Y. Yu H. Han X. Cheng W.. (2013). Vasa vasorum and plaque progression, and responses to atorvastatin in a rabbit model of atherosclerosis: contrast-enhanced ultrasound imaging and intravascular ultrasound study. Heart 99, 48–54. 10.1136/heartjnl-2012-302775 23100286

[B180] Tsaftaris S. A. Zhou X. Tang R. Li D. Dharmakumar R. (2013). Detecting myocardial ischemia at rest with cardiac phase-resolved blood oxygen level-dependent cardiovascular magnetic resonance. Circ. Cardiovasc. Imaging 6, 311–319. 10.1161/CIRCIMAGING.112.976076 23258476 PMC3684209

[B181] Ugander M. Bagi P. S. Oki A. J. Chen B. Hsu L. Y. Aletras A. H.. (2012). Myocardial edema as detected by pre-contrast T1 and T2 CMR delineates area at risk associated with acute myocardial infarction. JACC Cardiovasc. Imaging 5, 596–603. 10.1016/j.jcmg.2012.01.016 22698528 PMC3769169

[B182] Unger E. F. (2001). Experimental evaluation of coronary collateral development. Cardiovasc. Res. 49, 497–506. 10.1016/S0008-6363(00)00285-6 11166263

[B183] Urboniene D. Haber I. Fang Y. H. Thenappan T. Archer S. L. (2010). Validation of high-resolution echocardiography and magnetic resonance imaging vs. high-fidelity catheterization in experimental pulmonary hypertension. Am. J. Physiol. Lung Cell Mol. Physiol. 299, L401–L412. 10.1152/ajplung.00114.2010 20581101 PMC2951068

[B184] Verdouw P. D. van den Doel M. A. de Zeeuw S. Duncker D. J. (1998). Animal models in the study of myocardial ischaemia and ischaemic syndromes. Cardiovasc. Res. 39, 121–135. 10.1016/S0008-6363(98)00069-8 9764194

[B185] Viles-Gonzalez J. F. Poon M. Sanz J. Rius T. Nikolaou K. Fayad Z. A.. (2004). *In vivo* 16-slice, multidetector-row computed tomography for the assessment of experimental atherosclerosis: comparison with magnetic resonance imaging and histopathology. Circulation 110, 1467–1472. 10.1161/01.CIR.0000141732.28175.2A 15353509

[B186] Voeller R. K. Aziz A. Maniar H. S. Ufere N. N. Taggar A. K. Bernabe N. J. Jr.. (2011). Differential modulation of right ventricular strain and right atrial mechanics in mild vs. severe pressure overload. Am. J. Physiol. Heart Circ. Physiol. 301, H2362–H2371. 10.1152/ajpheart.00138.2011 21926343 PMC3233814

[B187] Vucic E. Dickson S. D. Calcagno C. Rudd J. H. F. Moshier E. Hayashi K.. (2011). Pioglitazone modulates vascular inflammation in atherosclerotic rabbits noninvasive assessment with FDG-PET-CT and dynamic contrast-enhanced MR imaging. JACC Cardiovasc. Imaging 4, 1100–1109. 10.1016/j.jcmg.2011.04.020 21999870 PMC3253377

[B188] Walmsley R. (1978). Anatomy of human mitral valve in adult cadaver and comparative anatomy of the valve. Br. Heart J. 40, 351–366. 10.1136/hrt.40.4.351 565642 PMC482803

[B189] Wang M. Baker L. Tsai B. M. Meldrum K. K. Meldrum D. R. (2005). Sex differences in the myocardial inflammatory response to ischemia-reperfusion injury. Am. J. Physiol. Endocrinol. Metab. 288, E321–E326. 10.1152/ajpendo.00278.2004 15367393

[B190] Watanabe Y. (1980). Serial inbreeding of rabbits with hereditary hyperlipidemia (WHHL-rabbit). Atherosclerosis 36, 261–268. 10.1016/0021-9150(80)90234-8 7406953

[B191] Webb S. Brown N. A. Anderson R. H. (1996). The structure of the mouse heart in late fetal stages. Anat. Embryol. 194, 37–47. 10.1007/BF00196313 8800421

[B192] Wetterholm R. Caidahl K. Volkmann R. Brandt-Eliasson U. Fritsche-Danielson R. Gan L. M. (2007). Imaging of atherosclerosis in WHHL rabbits using high-resolution ultrasound. Ultrasound Med. Biol. 33, 720–726. 10.1016/j.ultrasmedbio.2006.11.012 17383806

[B193] White F. C. Roth D. M. Bloor C. M. (1986). The pig as a model for myocardial ischemia and exercise. Lab. Anim. Sci. 36, 351–356. 3773444

[B194] Wichi R. Malfitano C. Rosa K. De Souza S. B. Salemi V. Mostarda C.. (2007). Noninvasive and invasive evaluation of cardiac dysfunction in experimental diabetes in rodents. Cardiovasc. Diabetol. 6:14. 10.1186/1475-2840-6-14 17462095 PMC1866223

[B195] Wiesmann F. Frydrychowicz A. Rautenberg J. Illinger R. Rommel E. Haase A.. (2002). Analysis of right ventricular function in healthy mice and a murine model of heart failure by *in vivo* MRI. Am. J. Physiol. Heart Circ. Physiol. 283, H1065–H1071. 10.1152/ajpheart.00802.2001 12181136

[B196] Worthley S. G. Helft G. Fuster V. Fayad Z. A. Rodriguez O. J. Zaman A. G.. (2000). Noninvasive *in vivo* magnetic resonance imaging of experimental coronary artery lesions in a porcine model. Circulation 101, 2956–2961. 10.1161/01.CIR.101.25.2956 10869269

[B197] Wu J. You J. Jiang G. Li L. Guan A. Ye Y.. (2012). Noninvasive estimation of infarct size in a mouse model of myocardial infarction by echocardiographic coronary perfusion. J. Ultrasound Med. 31, 1111–1121. 10.1136/heartjnl-2012-302920c.1 22733860

[B198] Xiangdong L. Yuanwu L. Hua Z. Liming R. Qiuyan L. Ning L. (2011). Animal models for the atherosclerosis research: a review. Protein Cell. 2, 189–201. 10.1007/s13238-011-1016-3 21468891 PMC4875304

[B199] Yanni A. E. (2004). The laboratory rabbit: an animal model of atherosclerosis research. Lab. Anim. 38, 246–256. 10.1258/002367704323133628 15207035

[B200] Young A. A. French B. A. Yang Z. Cowan B. R. Gilson W. D. Berr S. S.. (2006). Reperfused myocardial infarction in mice: 3D mapping of late gadolinium enhancement and strain. J. Cardiovasc. Magn. Reson. 8, 685–692. 10.1080/10976640600721767 16891227

[B201] Yu X. Tesiram Y. A. Towner R. A. Abbott A. Patterson E. Huang S.. (2007). Early myocardial dysfunction in streptozotocin-induced diabetic mice: a study using *in vivo* magnetic resonance imaging (MRI). Cardiovasc. Diabetol. 6:6. 10.1186/1475-2840-6-6 17309798 PMC1805425

[B202] Yuan L. Wang T. Liu F. Cohen E. D. Patel V. V. (2010). An evaluation of transmitral and pulmonary venous Doppler indices for assessing murine left ventricular diastolic function. J. Am. Soc. Echocardiogr. 23, 887–897. 10.1016/j.echo.2010.05.017 20591622 PMC2910830

[B203] Yue P. Arai T. Terashima M. Sheikh A. Y. Cao F. Charo D.. (2007). Magnetic resonance imaging of progressive cardiomyopathic changes in the db/db mouse. Am. J. Physiol. Heart Circ. Physiol. 292, H2106–H2118. 10.1152/ajpheart.00856.2006 17122193

[B204] Zaragoza C. Gomez-Guerrero C. Martin-Ventura J. L. Blanco-Colio L. Lavin B. Mallavia B.. (2011). Animal models of cardiovascular diseases. J. Biomed. Biotechnol. 2011:497841. 10.1155/2011/497841 21403831 PMC3042667

[B205] Zeng W. Wen X. Gong L. Sun J. Yang J. Liao J.. (2015). Establishment and ultrasound characteristics of atherosclerosis in rhesus monkey. Biomed. Eng. Online 14(Suppl. 1):S13. 10.1186/1475-925X-14-S1-S13 25602196 PMC4306102

[B206] Zhang Y. Takagawa J. Sievers R. E. Khan M. F. Viswanathan M. N. Springer M. L.. (2007). Validation of the wall motion score and myocardial performance indexes as novel techniques to assess cardiac function in mice after myocardial infarction. Am. J. Physiol. Heart Circ. Physiol. 292, H1187–H1192. 10.1152/ajpheart.00895.2006 17028161

[B207] Zhou R. Pickup S. Glickson J. D. Scott C. H. Ferrari V. A. (2003). Assessment of global and regional myocardial function in the mouse using cine and tagged MRI. Magn. Reson. Med. 49, 760–764. 10.1002/mrm.10423 12652548

[B208] Zhu Y. Carmeliet P. Fay W. P. (1999). Plasminogen activator inhibitor-1 is a major determinant of arterial thrombolysis resistance. Circulation 99, 3050–3055. 10.1161/01.CIR.99.23.3050 10368124

